# Comparative genomics of the *Smithellaceae*: reductive evolution and a mobile alkane-activation module in *Smithella*

**DOI:** 10.1093/ismeco/ycag275

**Published:** 2026-09-28

**Authors:** Boonfei Tan, Christian Zafra, Charmaine Ng

**Affiliations:** Institutional Research Office, Manila Central University, Caloocan City 1400, The Philippines; Department of Biology, College of Arts and Sciences, Manila Central University, Caloocan City 1400, The Philippines; Institutional Research Office, Manila Central University, Caloocan City 1400, The Philippines

**Keywords:** comparative genomics, *Smithellaceae*, *Smithella*, syntrophy, genome reduction, interspecies electron transfer, metabolic modelling

## Abstract

*Smithella* are repeatedly implicated as primary alkane activators in methanogenic hydrocarbon-degrading communities, yet the propionate-oxidizing type species and many relatives cannot activate alkanes, and the genus’s diversity and functional differentiation remain poorly resolved. Here, we present genus-scale comparative genomics of *Smithella* across 108 *Smithellaceae* metagenome-assembled genomes from three genera. The 74 *Smithella* genomes resolve into 23 species, 15 of them unnamed. Alkylsuccinate synthase occurs as two paralogous families, A and B. They differ by a third of their residues and coexist in the same genome. Both reject the species topology, family B decisively. *assA* has been gained and lost independently in several lineages. The families differ in mobility: family A is single-copy, confined to two species and in a conserved neighbourhood, whereas family B duplicates within genomes, spans four species and occupies a far less conserved context, most often beside a 2-hydroxyisobutanoyl-CoA mutase. Reductive specialization is pervasive, including genus-wide loss of flagellar motility, dissimilatory sulfite reduction, and *de novo* cobamide (vitamin B₁₂) biosynthesis. In consortium models, both lineages behaved equivalently on propionate, the substrate they share, and a hydrogenotrophic partner was obligatory for alkane oxidation, for which H₂ and formate are the only electron acceptors. The genus appears to favour propionate dismutation, thermodynamically preferred when hydrogen removal is incomplete; both lineages have largely lost the methylmalonyl-CoA epimerase most sister genomes retain. Alkane activation is therefore a mobile accessory on an otherwise conserved syntrophic core. Acquiring it widens the substrate range while tightening the requirement for a hydrogenotrophic partner.

## Introduction

Syntrophic bacteria catalyse thermodynamically unfavourable oxidations that proceed only when coupled to methanogenic partners maintaining low H₂ partial pressures [1, 2]. Genome sequencing and characterization of model syntrophs, including *Syntrophus aciditrophicus* [3], *Pelotomaculum thermopropionicum* [4, 5], and *Syntrophomonas wolfei* [6], have revealed a conserved template: streamlined energy metabolism, limited respiratory flexibility, and dependence on reverse electron transport [7]. Syntrophic consortia also depend on cross-feeding of vitamins and amino acids, most members being unable to synthesize essential cofactors independently [8–10]. Obligate interdependence extends beyond electron disposal to biosynthetic functions lost during adaptation to a partnered lifestyle.

Among syntrophic bacteria, *Smithellaceae* occupy a uniquely broad metabolic niche. The type species, *Smithella propionica* LYP^T^, dismutates propionate to acetate and butyrate [11, 12], and 16S rRNA surveys routinely detect *Smithella* as a dominant propionate oxidizer in anaerobic digesters [8, 13]. Yet *Smithella* is simultaneously the primary alkane activator in methanogenic hydrocarbon-degrading enrichments [14, 15] from petroleum reservoirs [16, 17], oil sands tailings ponds [18–21], and contaminated aquifers [22], where alkane oxidation proceeds via fumarate addition [15, 23]. Such enrichments often require months to years before methane production begins [18, 24, 25], and no alkane-degrading *Smithella* has been obtained in pure culture, consistent with strong partner dependence whose requirements remain unresolved.

Understanding of *Smithella* metabolism rests on a small number of cultivation-independent studies. Tan *et al.* [26, 27] identified the *assA* gene in draft genomes from two different methanogenic alkane-degrading cultures, Wawrik *et al.* [15] reconstructed electron transfer pathways from a *Smithella* sp. SDB scaffold, and Qin *et al.* [14] reported an Hdr/Mvh electron bifurcation complex and nitrogen fixation genes in Ca. *Smithella cisternae* M82_1. These studies characterized the metabolism of individual genomes but could not address population-level questions: how *assA* was acquired and why it has been lost repeatedly; how extensively genomes have been streamlined; and whether obligate partner dependence reflects biosynthetic deficiencies in cofactors such as B₁₂, heme, and the C1 carriers shared with methanogenic archaea [28], beyond thermodynamic constraints.

Here, we present a comparative genomic analysis of 108 *Smithellaceae* Metagenome-Assembled Genome (MAGs) spanning three genera (*Smithella*, *n* = 74; UBA8904, *n* = 26; MVRV01, *n* = 8), including 18 *assA*+ and 56 *assA*− *Smithella* genomes. We use phylogenomics, pangenome analysis, ancestral state reconstruction, and genome-scale metabolic modelling to characterize the genomic basis of syntrophic obligacy across the family. Beyond presence/absence, we use matched consortium models to test whether the *assA* split changes quantitative methanogenic performance.

## Methods

### Genome selection and phylogenomics

In total, 108 *Smithellaceae* MAGs, comprising 74 *Smithella* (18 *assA*+, 56 *assA*−), 26 UBA8904, and 8 MVRV01, plus the *S. aciditrophicus* SB outgroup (GCF_000013405.1) were analysed, all verified with CheckM2 [29] against GTDB r232 (completeness 57%–100%, median 95.5%; contamination ≤9.2%), with absence calls confirmed on a MIMAG high-quality subset ([30]; 55 *Smithella*, ≥90%/≤5%). We follow the GTDB circumscription, in which *Smithellaceae* comprises *Smithella*, UBA8904, and MVRV01, rooted by *Syntrophus*. The bac120 set was aligned with GTDB-Tk v2.6.1 (r232 reference package) [31], trimmed with trimAl [32] and analysed with IQ-TREE2 [33] (JTTDCMut+F+R6; 4737 positions; 1000 UFBoot + 1000 SH-aLRT). Pairwise Average Nucleotide Identity (ANI) from skani v0.3.1 [34] was clustered at 95.5% by single linkage, slightly above the 95% species boundary [35], and *assA* status determined by hmmsearch [36] against a curated AssA profile (Supplementary Methods).

### Comparative genomics

Protein-coding genes from Prokka [37] for the 109 validated genomes were clustered into 9913 orthogroups with OrthoFinder v2.5.5 [38] (Table S1) and annotated with eggNOG-mapper v2 [39] and InterProScan v5.75 [40]. Unannotated orthogroups were further characterized by genomic context and structure-based homology search (ESMFold [41]/Foldseek [42]/ColabFold [43]) and catalogued in Table S2. Pathway presence was scored per genome group as the percentage encoding at least one copy of the relevant orthogroup.

### Ancestral state reconstruction

Orthogroup presence/absence was reconstructed by Dollo parsimony [44, 45] on a species tree inferred from 914 single-copy orthogroups (Supplementary Methods) and, for sensitivity, by maximum-likelihood (Mk; castor [46]) and maximum parsimony, applied to the 9902 variable orthogroups, with stochastic character mapping in phytools [47] (Supplementary Methods). Full procedures are in Supplementary Methods.

### Genomic feature surveys

The *assA* genomic island, flagellar and chemotaxis genes, the conductive-pilus (e-pilin) potential of PilA (aromatic-density criteria of Walker *et al.* [48, 49] and Bray *et al.* [50]) and the direct interspecies electron transfer (DIET)-associated multiheme c-type cytochrome repertoire (references *Geobacter sulfurreducens* and *S. aciditrophicus* SB [49]) were characterized as in Supplementary Methods (Tables S3 and S4).

### Validated gene presence/absence

Absence calls were tested by profile-HMM search of the predicted proteomes (TIGRFAM/Pfam trusted cutoffs; hmmsearch --cut_ga) in high-quality genomes, validated by a between-genus positive control (the same HMMs in UBA8904 and MVRV01 at equal quality) and an internal control (the Type IV pilus system, recovered in all *Smithella*). Cofactor auxotrophy used the same framework (Tables S5B and S6). Flanking-synteny groups were compared by exact permutation over locus labels, since pairwise similarities share loci and are not independent.

### Genome-scale metabolic modelling

Pan-genome-scale models for the *assA*+ and *assA*− lineages were reconstructed from representative genomes with gapseq v1.4.0 [51] and gap-filled on a minimal anaerobic medium with the substrate as sole carbon source. The *assA*+ model additionally received a gene-supported alkane-activation module (hexadecane activation by fumarate addition, then mass- and charge-balanced oxidation of the alkylsuccinate to eight acetyl-CoA; 15,23), the only reactions added by hand; the two reconstructions otherwise differ in accessory content, reflecting their source genomes (Table 1). The comparison tests whether alkane degradation is a modular addition to a shared syntrophic core, by running both lineages on the ancestral substrate propionate and the *assA*+ model on hexadecane.

**Table 1:** Consortium modelling of the two *Smithella* lineages.

	*assA*+ / hexadecane	*assA*− / propionate	*assA*+ / propionate
Substrate	hexadecane (C_1 6_)	propionate (C_3_)	propionate (C_3_)
Alkane activation (AssA)	present	absent	present but unused
Grows on substrate	yes	yes	yes
CH₄ yield (% of Buswell maximum)	10.44 (85%)	1.22 (70%)	1.23 (70%)
Acetate:hydrogenotrophic partitioning	65:35	86:14	86:14
Curated reactions added (gene-supported)	AssA + balanced C_1 6_ oxidation to 8 acetyl-CoA (17 [2H])	none	none

Each lineage model runs in the same three-member consortium (*Smithella* with a hydrogenotrophic and an acetoclastic methanogen) on its substrate. On propionate, both lineages oxidize by dismutation and give near-identical outputs; the alkane-activation module is added only to the alkane-degrading model and used only on hexadecane. Methane yields are a fixed fraction of the Buswell theoretical maximum [58]; the remainder enters biomass. Partitioning follows from the degradation stoichiometry. Models are gap-filled to grow (Methods); cobalamin is the one *de novo* cofactor pathway lost, with menaquinone unresolved (Table S6).

For both lineages, the pan-GEM was embedded in the same minimal closed three-member consortium with a hydrogenotrophic *Methanocorpusculum*-type and an acetoclastic *Methanosaeta*-type sink, with H₂, formate, and acetate export blocked so products were consumed internally. Community behaviour was solved with the SteadyCom formulation [52] via COBRApy [53], which fixes no member abundances *a priori*, cross-checked against MICOM 0.37.1 [54], which agreed, and the two propionate-oxidation routes were compared thermodynamically following Dolfing [55] (Table S7). Models and scripts are deposited at Zenodo (https://doi.org/10.5281/zenodo.19148249).

## Results

### Phylogenomic framework and genus-level diversification

A maximum-likelihood phylogenomic tree from the bac120 marker gene set [56] resolves three genera: *Smithella* (74 genomes), UBA8904 (26 genomes), and MVRV01 (8 genomes) (Fig. 1; Table S8). ANI analysis (single-linkage clustering at 95.5% identity) resolves the 74 *Smithella* into 23 species (Fig. S1). Fifteen clusters contain no genome with a GTDB r232 species assignment and fall below 95% ANI against every named genome. They represent lineages without a named GTDB species; 12 are single genomes (Table S8). Previously published MAGs [14, 15, 26, 27, 57] fall into three species, all *assA*+. The 18 *assA*+ genomes span five species, interspersed with *assA*− genomes, although 13 of the 18 fall within one species. Apparent gene content is strongly confounded by genome completeness (r = 0.70; Fig. 2A). Diversity was therefore assessed among high-quality genomes only. Even then the pan-genome is open: the core comprises 11% of orthogroups, and mean between-species gene-content similarity is a Jaccard of 0.51 (Fig. 2B and C). Bulk gene content is structured predominantly by species (PERMANOVA pseudo-F = 9.1, *P* = .001) rather than by *assA* status, and the residual *assA* signal is weak (species-level pseudo-F = 1.7, *P* = .04); *assA*+ species are interspersed among *assA*− species in ordination (Fig. 2C). The 2741 species-restricted orthogroups (37% of the pan-genome) are dominated by genes of unknown function (74% hypothetical), only ~10% assignable to metabolism (Fig. 2D; Table S9).

**Figure 1:**
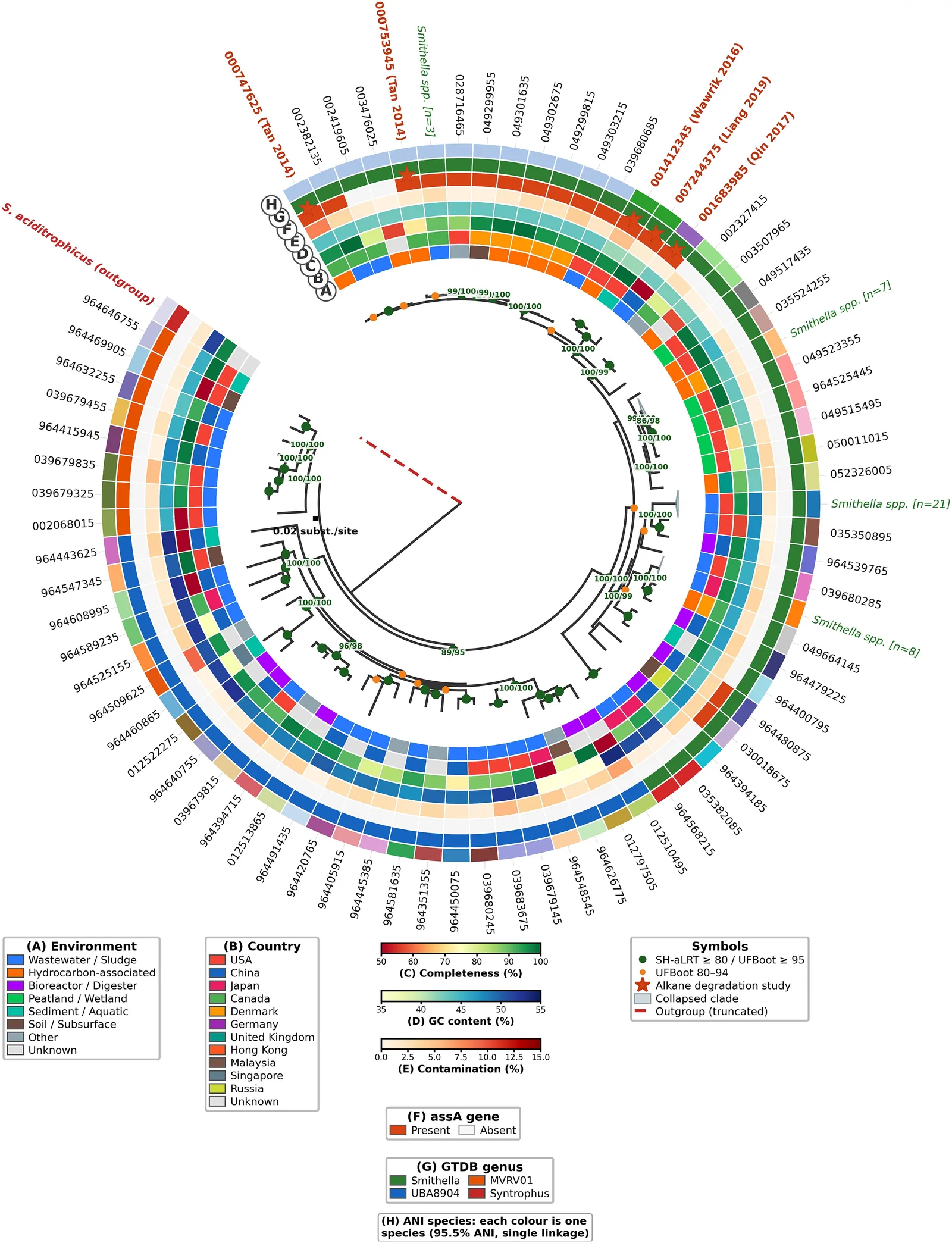
Phylogenomic tree of 108 *Smithellaceae* plus *Syntrophus* outgroup (109 genomes). Maximum-likelihood phylogeny inferred from the concatenated bac120 marker gene alignment (4737 amino acid positions) using IQ-TREE2 [33] under the JTTDCMut+F+R6 model. The tree is rooted on *S. aciditrophicus* SB (GCF_000013405.1), which lies in the sister family *Syntrophaceae* under GTDB taxonomy (a valid outgroup to the *Smithellaceae* ingroup; see Methods). Branch support values are shown as SH-aLRT (%) / UFBoot (%) from 1000 replicates each. Concentric rings show, from the innermost outwards, isolation environment (A), country of origin (B), CheckM2 completeness (C), GC content (D), contamination (E), *assA* presence or absence (F), GTDB genus (G), and ANI cluster (H; single-linkage clustering at 95.5% ANI, each colour one cluster: 23 species among the 74 *Smithella* genomes, 56 clusters across all 109; Table S8). Red stars mark the genomes characterized in previous alkane-degradation studies. Tip labels are GenBank assembly accessions with the GCA_ prefix and version suffix omitted; full accessions are in Table S8. The three *Smithellaceae* genera form well-supported monophyletic clades. Within *Smithella*, *assA*+ genomes (encoding alkylsuccinate synthase, ring F) are distributed across multiple species-level clusters interspersed with *assA*− genomes, consistent with horizontal acquisition, intra-genus redistribution and repeated loss.

**Figure 2:**
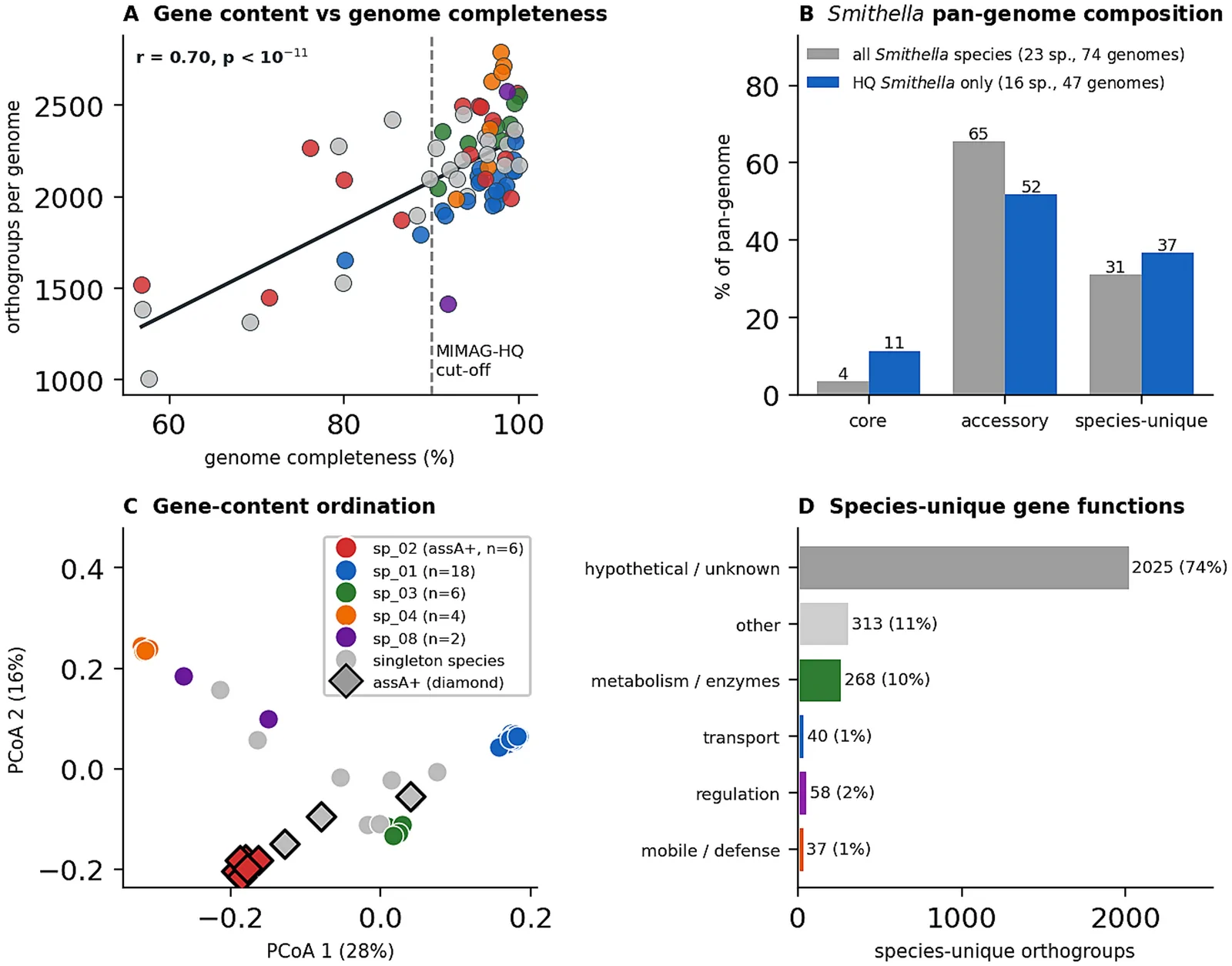
Genomic diversity among the *Smithella* species. (A) Apparent gene content is confounded by genome completeness (orthogroups per genome versus CheckM2 completeness, with the five multi-genome species coloured as in (C) and all remaining genomes grey; r = 0.70, *P* < 10^−11^; dashed line, MIMAG high-quality cut-off). Pan-genome analyses are therefore restricted to high-quality genomes. (B) Species-level pan-genome composition, all species versus high-quality species only: the core rises from 4% to 11% of orthogroups once incomplete genomes and species are excluded, while the pan-genome remains open (52% accessory, 37% species-restricted). (C) Principal-coordinates ordination of gene content (Jaccard distance) across the 47 high-quality *Smithella* genomes (≥90% completeness, ≤5% contamination, and contig N50 ≥ 10 kb; 16 species). This filter is stricter than the *n* = 55 MIMAG high-quality subset used for the absence analyses, in additionally requiring contig N50 ≥ 10 kb, and 9 of the 18 *assA*+ genomes pass it. The five well-sampled species (≥2 genomes) form distinct clusters (coloured), embedded in a continuum of singleton species (grey). *assA*+ genomes (diamonds) are scattered across four species rather than forming a separate group. (D) Functional categories of the 2741 species-unique orthogroups (Table S9). Gene content is structured predominantly by species and not by *assA* status (PERMANOVA; main text).

### Metabolic capacity across lineages

Comparative analysis of 9913 orthogroups revealed metabolic specialization and genome streamlining in *Smithella* (Table 2; Fig. S2), the most reduced of the three genera (mean 2.70 Mb / 2660 coding sequences, versus 2.85 Mb / 2829 for UBA8904, and 3.14 Mb / 3067 for MVRV01; Table S8). Central carbon metabolism is unevenly conserved: *fadA* and *fadB* were detected in 97% and 96% of genomes and the tricarboxylic acid cycle (TCA) steps *gltA* and *icd* in 97% and 88%, but fumarate reductase (*frdB*) in only 12%. The Wood–Ljungdahl pathway is absent from the genus altogether: no *Smithella* genome encodes carbon monoxide dehydrogenase (0 of 74), against 62% of MVRV01, and formyl-tetrahydrofolate synthetase is present in only 7%, against 42%–100% in the sister genera and the outgroup. The *acsA* orthogroups detected in 77% of genomes encode acetyl-CoA synthetase (EC 6.2.1.1), not the acetyl-CoA synthase (EC 2.3.1.169) of that pathway.

**Table 2:** Metabolic capacity across *Smithellaceae* and the *Syntrophus* outgroup (GTDB r232).

Gene or pathway	*Smithella* (*n* = 74)	UBA8904 (*n* = 26)	MVRV01 (*n* = 8)	*Syntrophus* (*n* = 1)
**Carbon activation**				
*assA* (alkylsuccinate synthase)	24	0	0	0
*bssE/bssD* (GRE activating enzyme)	28	12	62	0
*fadA* (β-oxidation thiolase)	97	69	25	0
*fadB* (enoyl-CoA hydratase)	96	92	25	0
*gltA* (citrate synthase)	97	85	100	100
*icd* (isocitrate DH)	88	88	100	100
*frdB* (fumarate reductase)	12	0	0	0
*porA/B* (pyruvate:Fd OR)	96	100	100	100
*korA/B* (2-oxoglutarate:Fd OR)	95	100	100	100
*cooS/cdhA* (CO-DH)	0	4	62	0
*acsA* (acetyl-CoA synthetase)	77	88	100	100
**Syntrophic electron transfer**				
*fdhA* (formate DH)	92	100	100	100
[FeFe]-Hase (*hyd/hnd*)	99	100	100	100
*etfA/B* (bifurcating)	96	100	100	100
*rnfA–G* (Rnf complex)	0	0	12	100
*nfnA/B* (NADH:Fd transhydrogenase)	0	0	0	0
*pta* (phosphotransacetylase)	86	15	100	0
*ackA* (acetate kinase)	54	69	88	0
**Terminal e** ^**-**^ **acceptors**				
***dsrAB* (sulfite reductase)**	**1**	**54**	**25**	**0**
*aprA/sat* (sulfate reduction)	0	0	0	0
*narG/napA* (nitrate reductase)	0	0	0	0
*nirS/nirK* (nitrite reductase)	0	0	0	0
*nrfA* (cyt *c* nitrite)	7	58	25	100
**Motility**				
***fliG* (flagellar switch)**	**0**	**50**	**100**	**100**
***motA/B* (flagellar motor)**	**0**	**46**	**100**	**100**
***flhA* (flagellar export)**	**0**	**46**	**100**	**100**
*cheA/cheY* (chemotaxis)	81	96	100	100
**Respiratory chain**				
*nuoA–N* (Complex I)	5	4	38	0
*petAB/qcrAB* (cyt bc₁)	89	27	88	100
*atpA–H* (F₁F₀ ATP synthase)	99	100	100	100
*menA–F* (menaquinone; any of six)	100	100	100	100
*menB* (committed step)	28	27	25	100
**Transport**				
*feoB* (Fe^2+^)	97	100	100	100
*nikMN* (nickel)	93	50	75	0
*cbiO* (cobalt/B₁₂)	85	0	100	100
*cysA/T* (sulfate)	85	96	100	100
*dctA/M* (C4-dicarboxylate)	99	100	100	100
*livF/H* (branched-chain AA)	99	100	100	100
*pstB/C* (phosphate)	96	92	100	100
*corA* (Mg^2+^)	95	96	100	100
*nrgA* (ammonium)	93	81	100	100
*oprF* (porin)	100	100	100	100
**Cofactor biosynthesis**				
**B₁₂ *de novo* (*cbiA/L*)**	**1**	**54**	**88**	**0**
B₁₂ salvage (*cobS/T/U*)	42	73	88	0
*hemA–C* (heme, early)	99	96	100	100
*hemH* (ferrochelatase)	0	0	0	0
*hemN* (coproporphyrinogenase)	0	0	0	0
*ahbA/D* (heme, alt. route)	93	88	100	100
*folA* (DHFR)	0	0	0	0
*folK/P/C* (folate core)	99	100	100	100
*bioB* (biotin synthase)	89	100	100	100
*bioF/D* (biotin upstream)	42	77	38	100
*thiE/G/S* (thiamine)	100	88	100	100
*ribD/E* (riboflavin)	97	100	100	100
*moaA–E* (molybdopterin)	99	100	100	100
**IET: H₂ase + FDH**				
*hydB* ([FeFe]-Hase)	99	96	100	100
*hndA* ([FeFe]-Hase)	99	100	100	100
*hndD* (NADP-red. Hase)	68	54	100	100
*fdhF* (formate DH)	80	96	100	100
*fdhA*-α (one paralogue)	74	81	88	100
*ttrB* (FDH FeS)	89	92	88	100
**Electron bifurcation**				
*etfA* (ETF α)	69	85	100	100
*etfB* (ETF β)	65	77	100	100
*bcd* (butyryl-CoA DH)	99	96	100	100
HdrABC/Mvh (bifurc.)	89	92	100	100
**Central metabolism**				
*porA* (PFOR α)	92	100	100	100
*porB* (PFOR β)	88	92	100	100
*mmdA* (MMCoA decarboxylase)	92	96	100	100
*sdhE* (MQ:fumarate reductase anchor)	73	73	100	0
**Accessory e** ^**-**^ **transfer (variable)**				
*qrcC/D* (MQ:Fd OR)	4	54	25	0

The *dsrAB* row is scored by profile-HMM (hmmsearch --cut_ga) because the annotation survey labels these genes *dsvA/dsvB* and underdetects the divergent sister copies; all other rows are scored by annotation. *assA* is scored on the curated HMM/tBLASTn call ([62]; 18 of 74). The single *dsrAB* and *cbiA* detections in *Smithella* both fall in genomes below the 90% completeness threshold. Rows reading zero in all four groups, including the free-living outgroup, indicate that no orthogroup carries that gene symbol in this catalogue; the function may still be present. *folA* is the clear case, since the folate core is present in 99%–100% of genomes. Such rows are not counted as losses and are not marked. Rows naming a complex or a gene set score the presence of any one member and therefore read at or above the single-gene rows beneath them (*porA, porB, hydB, hndA, menB*), which are scored individually. Values are the percentage of genomes in each group encoding at least one copy of the relevant orthogroup, scored over all genomes. Rows are grouped by functional module; genes in bold are the sister-validated reductive losses in *Smithella*.

Syntrophic electron disposal, by contrast, is near-universal: *fdhA* (92%), [FeFe]-hydrogenases (*hydB*; 99%), and electron-transferring flavoproteins (*etfB*; 96% across its two orthogroups, 65% for one alone as in Table 2) [59] were each conserved in >90% of genomes. Terminal electron acceptors are correspondingly sparse: the sulfate-activating enzymes (*sat*, *aprAB*) and the respiratory nitrate and nitrite reductases (*narG*/*napA*, *nirS*/*nir*K) are undetected in all four groups, while the cytochrome *c* nitrite reductase *nrfA* is sporadic in *Smithella* (7%) but common in UBA8904 (58%) and present in the outgroup. No lineage here encodes a complete dissimilatory sulfate-, nitrate-, or nitrite-reduction pathway. The one dissimilatory terminal reductase that varies is the sulfite reductase *dsrAB*, retained in the sister genera (UBA8904 53%, MVRV01 29% of high-quality genomes by profile-HMM; Table S5A) but absent from all 55 high-quality *Smithella* genomes, and therefore the only terminal-acceptor loss validated against the sister genera (Table 2). The 8-methylmenaquinol:fumarate reductase anchor (*sdhE*) is absent from 5 of the 18 *assA*+ genomes (28%); contrary to the absence reported for Ca. *S. cisternae* M82_1 [14], that genome encodes it. Rnf was detected in only one MVRV01 genome and the *Syntrophus* outgroup (2 of 109) and in no *Smithella* genome, whereas the HdrABC/Mvh electron-bifurcating complex is near-universal (89% of *Smithella* and 92%–100% of the sister genera; Table 2). Set against this retained flavin-based bifurcation machinery (*etfAB* and HdrABC/Mvh), the absence of Rnf suggests that *Smithella* recycles reduced ferredoxin and drives reverse electron transport through electron bifurcation or confurcation rather than an Rnf complex, unlike its Rnf-carrying relatives [7, 60].

Cofactor biosynthesis is intact except for cobalamin and, less certainly, menaquinone: the *de novo* cobalamin (B₁₂) route lacks the anaerobic corrin-ring steps. *Smithella* therefore appears unable to synthesize cobalamin *de novo* and depends on an external B₁₂ source. No other cofactor pathway is affected: the classical protoporphyrin-dependent heme route is also incomplete (*hemH* and *hemN* undetected under these gene names in all four groups, including the outgroup). Heme biosynthesis is retained through the alternative siroheme-dependent (Ahb) route [61] (*ahbA* and *ahbD* in over 90% of genomes) and the folate core (*folK*, *folP*) is intact (Table 2; Table S10). The sister genera retain the corrin-ring genes, making incompleteness an unlikely explanation for their absence in *Smithella* (Table S5A).

The *assA* gene was present in 18 of the 74 *Smithella* genomes and absent from all other genera (Fig. S3). Fisher’s exact tests across the 7973 orthogroups variable within *Smithella* identified 292 whose prevalence differed significantly between the 18 *assA*+ and 56 *assA*− genomes (Bonferroni), 204 more prevalent in *assA*+ and 88 in *assA*−; the most strongly enriched are the alkane-activation operon itself (Table S11).

### Gene gain and loss across the *Smithellaceae* phylogeny

Reconstruction across the 9902 variable orthogroups is strongly loss-biased under Dollo parsimony (single gain, no re-gain), at 15 losses per gain. Methods that permit re-gain give much weaker signals: 1.8 losses per gain under an asymmetric Mk model, and 1.8 gains per loss under maximum parsimony. The magnitude of the loss bias is therefore sensitive to whether re-gain is allowed. By Dollo, the *Smithella* stem shows net reduction (−500 orthogroups) and terminal branches average 561 losses per genome, or 404 above 99% completeness (Fig. 3C); all three descendant lineages underwent net reduction from the *Smithellaceae* ancestor’s repertoire. Terminal counts scale with genome completeness and are therefore upper bounds, but the direction of change is the same at every threshold.

**Figure 3:**
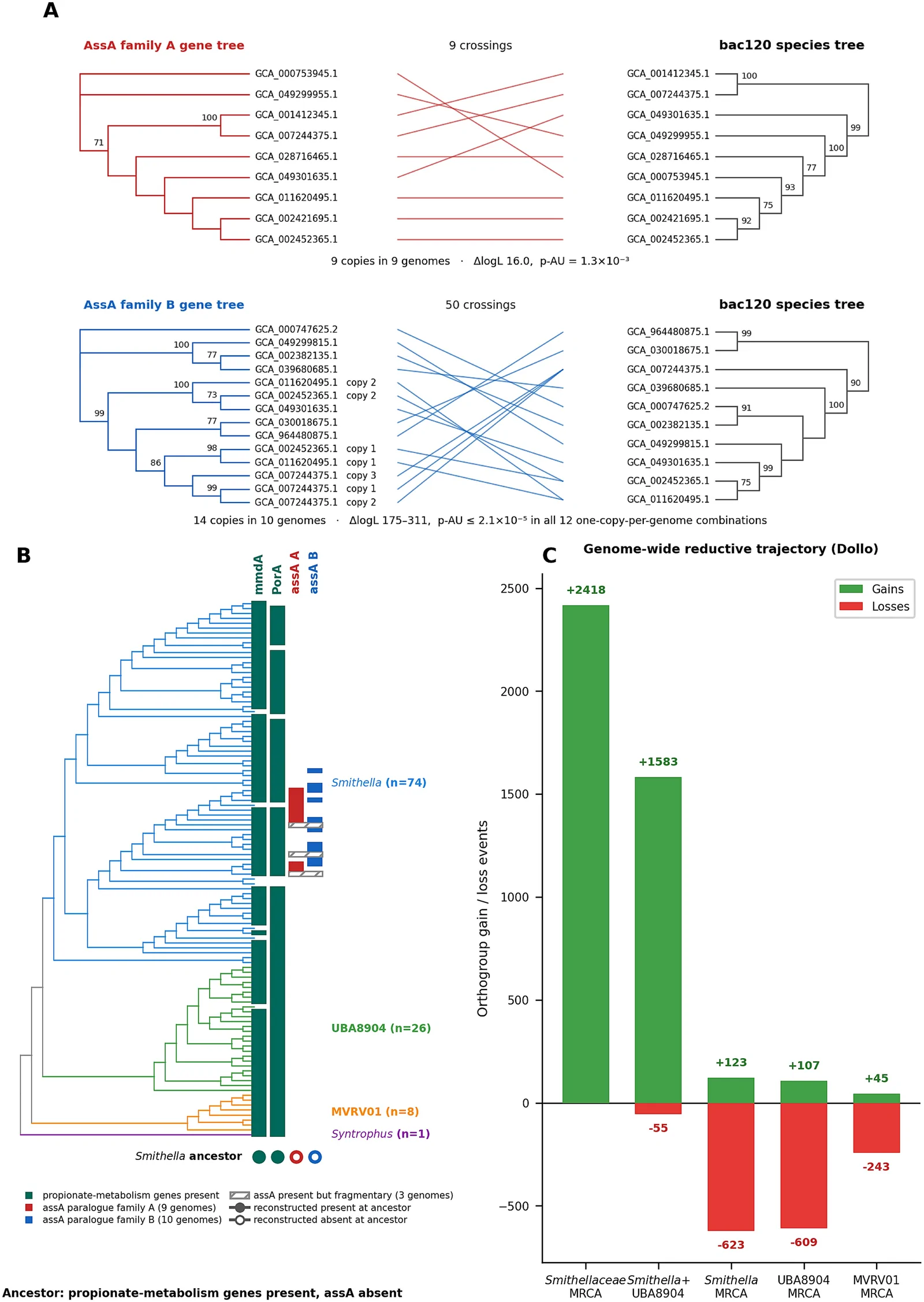
Horizontal transfer and intra-genus redistribution of *assA* in *Smithella*. (A) Tanglegrams comparing each AssA paralogue family’s maximum-likelihood protein tree (left) with the corresponding bac120 species tree (right), built separately for family A (9 copies in 9 genomes) and family B (all 14 copies in 10 genomes) so that paralogues are never mixed. Links join the same genome in the two trees, and their crossings mark topological conflict. Each family rejects the species topology on its own (family A p-AU = 1.3 × 10^−3^, ΔlogL 16.0; family B p-AU ≤ 2.1 × 10^−5^, ΔlogL 175–311 across all 12 one-copy-per-genome combinations); node labels give UFBoot support where it reaches 70. This indicates that *assA* has been redistributed among *Smithella* lineages by intra-genus horizontal transfer rather than inherited vertically. (B) Cladogram of 108 *Smithellaceae* plus the *Syntrophus* outgroup (109 genomes), branches coloured by genus (*Smithella* blue, *n* = 74; UBA8904 green, *n* = 26; MVRV01 orange, *n* = 8; *Syntrophus* purple, *n* = 1). Presence strips show the propionate-metabolism genes methylmalonyl-CoA decarboxylase (*mmdA*) and pyruvate:ferredoxin oxidoreductase (PorA), and alkylsuccinate synthase scored directly on the AssA proteins and split by paralogue family (*assA* A, *assA* B). Hatching marks the three genomes whose only AssA is too fragmentary to assign to a family. Circles below give the state reconstructed at the *Smithella* most recent common ancestor, concordant across maximum-likelihood, maximum-parsimony, and outgroup criteria, with the propionate-oxidation genes present (filled) and *assA* absent (open). *assA* is interspersed within *Smithella* subclades (topology only, branch lengths not to scale). (C) Orthogroup gain and loss events at key ancestral nodes (Dollo parsimony), showing the burst of +2418 orthogroups assigned to the *Smithellaceae* MRCA, which under Dollo is the reconstructed ancestral repertoire rather than a burst of gains, a net gain at the shared *Smithella*–UBA8904 ancestor, and net reduction at each of the three genus ancestors.

### Paralogous *assA* families and a mobile alkane-activation module

*assA* in *Smithella* is a single coherent AssA type with a horizontally dynamic history. All *Smithella* AssA proteins group to the exclusion of BssA, NmsA, and the PflB outgroup, and the high within-clade identity (at least 66%, mean 79% between genomes, against ~30% between distinct glycyl-radical enzymes) indicates these are all AssA rather than several unrelated enzymes (Fig. S4). The clade also contains the AssA of *Desulfatibacillum alkenivorans* AK-01, *Azoarcus* sp. HxN1, and *Desulfosarcina* sp. BuS5, 60%–73% identical to the *Smithella* proteins and overlapping the 66%–100% spread within *Smithella*. *Smithella* AssA is therefore not resolvably distinct from that of other genera. Its coding sequence is modestly GC-richer than its host genome (mean +8.0, range +4 to +12 across the 23 near-full-length copies in 15 genomes), consistent with, but not diagnostic of, a foreign origin.

The *Smithella* copies are two paralogous AssA families rather than alternative alleles: 94%–100% and 80%–99% identical internally but only 66%–70% to each other, placed at separate positions in the AssA phylogeny (Fig. S4). Family A (9 copies in 9 genomes) resolves as a single clade (SH-aLRT 100/UFBoot 100), whereas family B (14 copies in 10 genomes) falls into three well-supported groups. Those groups remain one family: they are 80%–91% identical to each other and each carries *ass*-island genes absent from family A. Four genomes carry both. Both are catalytically intact: the glycyl-radical glycine and the catalytic cysteine are conserved in every copy long enough to span them (Fig. S4), and the two alkane-degrading enrichments reported earlier [26, 27] contributed one genome of each family. This duplication is analogous to the *assA1/assA2* pair of *Desulfatibacillum* AK-01 but not orthologous to it: both *Smithella* families are closer to AK-01 AssA1 (73% and 70% identity) than to AssA2 (70% and 65%). The gene-tree versus species-tree comparison, repeated within each family, rejects the species topology in both (approximately unbiased test; family A p-AU = 1.3 × 10^−3^, ΔlogL 16.0; family B p-AU ≤ 2.1 × 10^−5^, ΔlogL 175–311 across all 12 one-copy-per-genome combinations; Fig. 3A; Table S12). The incongruence is therefore not an artefact of mixing paralogues. Homologous recombination among close relatives produces similar discordance, but would not generate two families differing by a third of their residues in disjoint neighbourhoods, and incomplete lineage sorting is unlikely across families that coexist in one genome. Reconstruction under an asymmetric Mk model (500 stochastic maps) favours gains and losses in several lineages (medians 5 and 7 across the 500 maps; probability of a single gain 0.01, and no map without a loss). Within family B, the copies held by one genome are generally not each other’s closest relatives. In two genomes, the pair falls in different, well-supported parts of the tree (UFBoot 98 and 100), implying separate acquisitions rather than duplication in place. Two of the three copies in GCA_007244375.1 are sisters (UFBoot 99), consistent with tandem duplication.

Mobile-element genes are not enriched around the locus: 4 occur within ±20 kb of the 23 *assA* copies against 4.1 expected from the genome-wide rate (annotation-independent Pfam-HMM, Poisson *P* = .58; Table S13). The assemblies are fragmented and insertion sequences often end contigs, which does not exclude a resident element. Both families carry the alkylsuccinate synthase holoenzyme operon: the catalytic alpha subunit with the beta (OG0002508, Dalk_1729) and gamma (OG0002507, Dalk_1730) subunits and a MasE-like protein. It is the operon, not *assA* alone, that has moved. Beyond the operon, the families differ categorically. Every gene of the *Desulfatibacillum* AK-01 *ass* loci and the *Azoarcus* HxN1 *mas* operon was aligned against the *Smithella* orthogroups (≥30% of self-score). Family B loci carry 12 orthogroups homologous to AK-01 *ass*-island genes: the glycyl-radical activating enzyme, the AssE chaperone, a carboxyl transferase, MmgE/PrpD, enoyl-CoA hydratases, and the B₁₂-dependent 2-hydroxyisobutanoyl-CoA mutase (*hcmAB*) with its *argK* chaperone. The mutase is the commonest of them, at 8 of 14 family B loci against 7 for the activating enzyme, and sits beside the operon as at AK-01 *ass* locus I, the candidate carbon-skeleton rearrangement that follows fumarate addition. Every one is absent from all nine family A loci. Family A loci instead sit among genes with no homologue at either AK-01 locus (*ndvB*, *rpoH*, *dctP*, *mce2R*, *atoC*). Flanking-gene similarity accordingly averages 0.46 within family A but 0.18 within family B (Jaccard, over the 19 of 23 loci with at least five flanking genes recovered; exact permutation *P* = 5 × 10^−5^), with 0.08 between families. Family B has therefore retained the ancestral *ass* island, whereas family A retains only the operon and sits in an unrelated chromosomal neighbourhood (Fig. 4). Family A’s narrower host range and the absence of a phylogenetic control on the permutation test both qualify that comparison; horizontal movement without a resident element is consistent with the data but not established by it. *assA* is present in only 5 of 23 species, interspersed within *Smithella* subclades (Fig. 3B), and tBLASTn finds no full-length *assA* and no island remnant in *assA*− genomes (Fig. S3). Ancestral-state reconstruction indicates the polarity: methylmalonyl-CoA decarboxylase (*mmdA*, 92%) and pyruvate:ferredoxin oxidoreductase (92%) are reconstructed as present at the *Smithella* ancestor by both maximum-likelihood and maximum-parsimony and retained across both sister genera and the outgroup. *assA* is reconstructed as absent by all methods and from every sister lineage (Fig. 3B; Table S14; from the co-located OG0002508 marker, which tracks *assA* in 17 of 18 *assA*+ and 1 of 56 *assA*− genomes). One species contains both *assA*+ and *assA*− genomes. The polarity within it is therefore not assumed. The genus ancestor is reconstructed as lacking *assA*; the simplest reading is later acquisition of alkane activation spread by horizontal transfer, though an ancestral origin followed by loss cannot be excluded.

**Figure 4:**
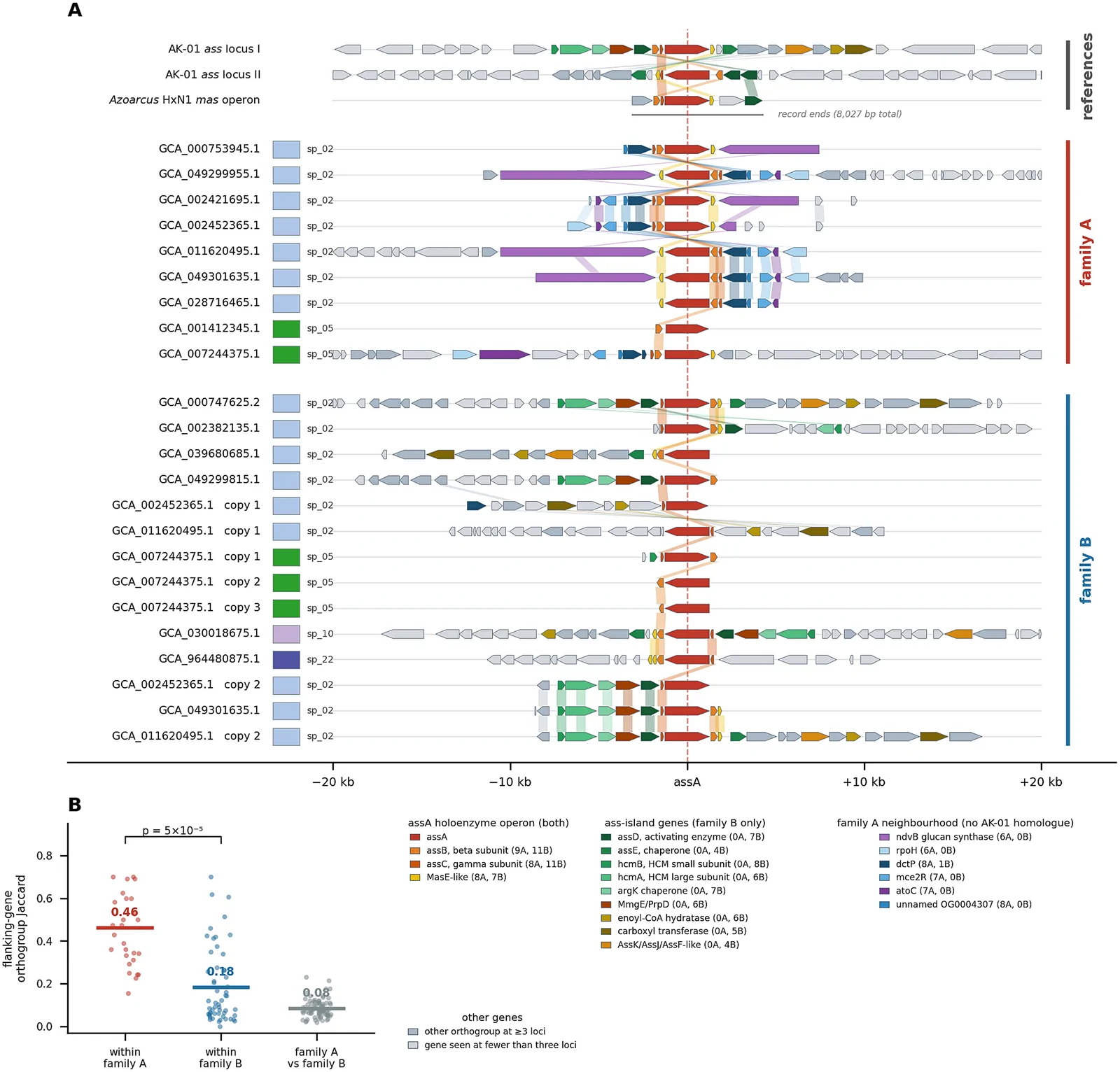
Flanking-gene context of the two *assA* paralogue families. (A) Genomic context around all 23 *assA* copies (±20 kb), with the two *Desulfatibacillum* AK-01 *ass* loci and the *Azoarcus* HxN1 *mas* operon as references at the top. The HxN1 record is shorter than ±20 kb; its track is marked where the record ends. One row per copy ordered to match the family gene trees of Fig. 3A and labelled by paralogue family, which makes the four genomes carrying both families appear more than once; genes are coloured by orthogroup and grouped by role, and ribbons link shared orthogroups between adjacent rows. Red and orange mark the *assA* holoenzyme operon carried by both families (the catalytic alpha subunit, the beta subunit OG0002508 and the gamma subunit OG0002507, and the MasE-like OG0002345). Blues and purples mark the family A neighbourhood [*mce2R*, *atoC*, *rpoH*, *dctP*, the *ndvB*-like cyclic β-1,2-glucan synthase (~8.7 kb) and the unnamed OG0004307], absent from every family B locus except *dctP*, which occurs at one. Greens and olives mark the family B neighbourhood, absent from every family A locus: the activating enzyme *assD*, the chaperone *assE*, the 2-hydroxyisobutanoyl-CoA mutase subunits *hcmA* and *hcmB* with their *argK* chaperone, MmgE/PrpD, an enoyl-CoA hydratase, a carboxyl transferase, and an AssK/AssJ/AssF-like protein. Slate grey marks orthogroups shared across at least three loci, pale grey those at fewer. The key names 9 of the 12 family-B-only orthogroups homologous to AK-01 *ass* locus I; the other three fall in the shared-orthogroup grey. The swatch and sp_NN label beside each track give the ANI species of that genome, coloured as in Fig. 1 (ring H): family A is confined to two species (sp_02, sp_05), whereas family B spans four (sp_02, sp_05, sp_10, sp_22). The fifth *assA*-bearing species carries only a fragmentary copy and is not assigned to a family, which is why four species appear here against five in the Results. (B) Every pairwise flanking-gene orthogroup similarity, one point per pair of loci, with group means marked. Flanking-gene orthogroup similarity averages 0.46 within family A but only 0.18 within family B (Jaccard, over the 19 of 23 loci with at least five flanking genes recovered; exact permutation *P* = 5 × 10^−5^; between-family 0.08). Family A therefore sits in a conserved neighbourhood, and family B does not. Two caveats apply: family A’s narrower host range (two species against four) may contribute, and the permutation test does not control for host phylogeny.

The syntenic *ass* operon carries *assA* with the beta and gamma subunits *assB* and *assC* and a MasE-like protein; *assD* and *assE* occur only at family B loci (Fig. 4). The operon orthogroups are present in 94%–100% of *assA*+ genomes (Fig. 4). Of the 22 orthogroups in the *assA*-island neighbourhood (Table S15), only a universal stress protein (UspA2, 13 of 18 versus 0 of 56) is among the 292 *assA*-associated orthogroups; an adenylate kinase and a *p*-benzoquinone reductase are enriched only nominally (Table S15). These lie away from the operon and are more plausibly co-selected with alkane degradation than co-transferred as a block.

### Loss of motility and chemotaxis

A prominent genomic signature of syntrophic specialization is the loss of flagellar motility in all 74 *Smithella* genomes: not a single flagellar structural, motor, or export apparatus gene was detected (median CheckM2 completeness 96%), regardless of *assA* status (Table S16). Flagellar genes are still detected in the sister genera (UBA8904 38%–50% across the genes of Table S16, MVRV01 100%), making a technical artefact unlikely. The apparatus is absent as a block of ~30 genes, which assembly dropout would not produce, and in UBA8904, it was lost independently in 4 to 5 events. Dollo parsimony reconstructs the *Smithella* loss as a single deletion at the genus most recent common ancestor (MRCA), and the absence of pseudogenes in the most complete genomes (*n* = 12 at ≥99%) supports genuine deletion. Throughout, a gene is counted as lost only where the same profile-HMM detects it in the sister genera at equal completeness and the absence persists in the most contiguous genomes; flagellar loss meets both (contig N50 up to 233 kb). Chemotaxis genes are more depleted in *assA*+ than *assA*− *Smithella* (*cheA* 44% versus 89%, *cheB* 33% versus 89%, *cheR* 28% versus 88%), but that comparison is sensitive to the higher fragmentation of *assA*+ assemblies and is not counted among the validated losses.

### The propionate route and its consequences for partner dependence

Matched consortium models were reconstructed for the two lineages as a stoichiometric check on the gene-content results. They were built independently and differ in accessory reactions (Table 1). The comparison is therefore not a single-gene knockout. Both lineages are obligate syntrophs that cannot grow in pure culture and require a hydrogen- and formate-consuming partner [1, 11], a thermodynamic property the stoichiometric models do not themselves enforce [63].

On the shared substrate, the two lineages were metabolically near-identical: methane yields at the same fraction of the Buswell maximum (~70% on propionate, the remainder entering biomass) and the same predominantly acetoclastic partitioning, ~86:14 (Table 1; Fig. 5). Propionate is modelled by the dismutation route characterized for the type species *S. propionica* [11]. Methane arose acetoclastically and hydrogenotrophically, matching fatty-acid- and alkane-degrading enrichments [14, 15, 18–20]; only the total hydrogenotrophic fraction is reported, because its split between H₂ and formate is not identifiable from the community stoichiometry. Given hexadecane, the *assA*+ model routed the substrate through the alkane-activation module into the same central metabolism (Table 1).

**Figure 5:**
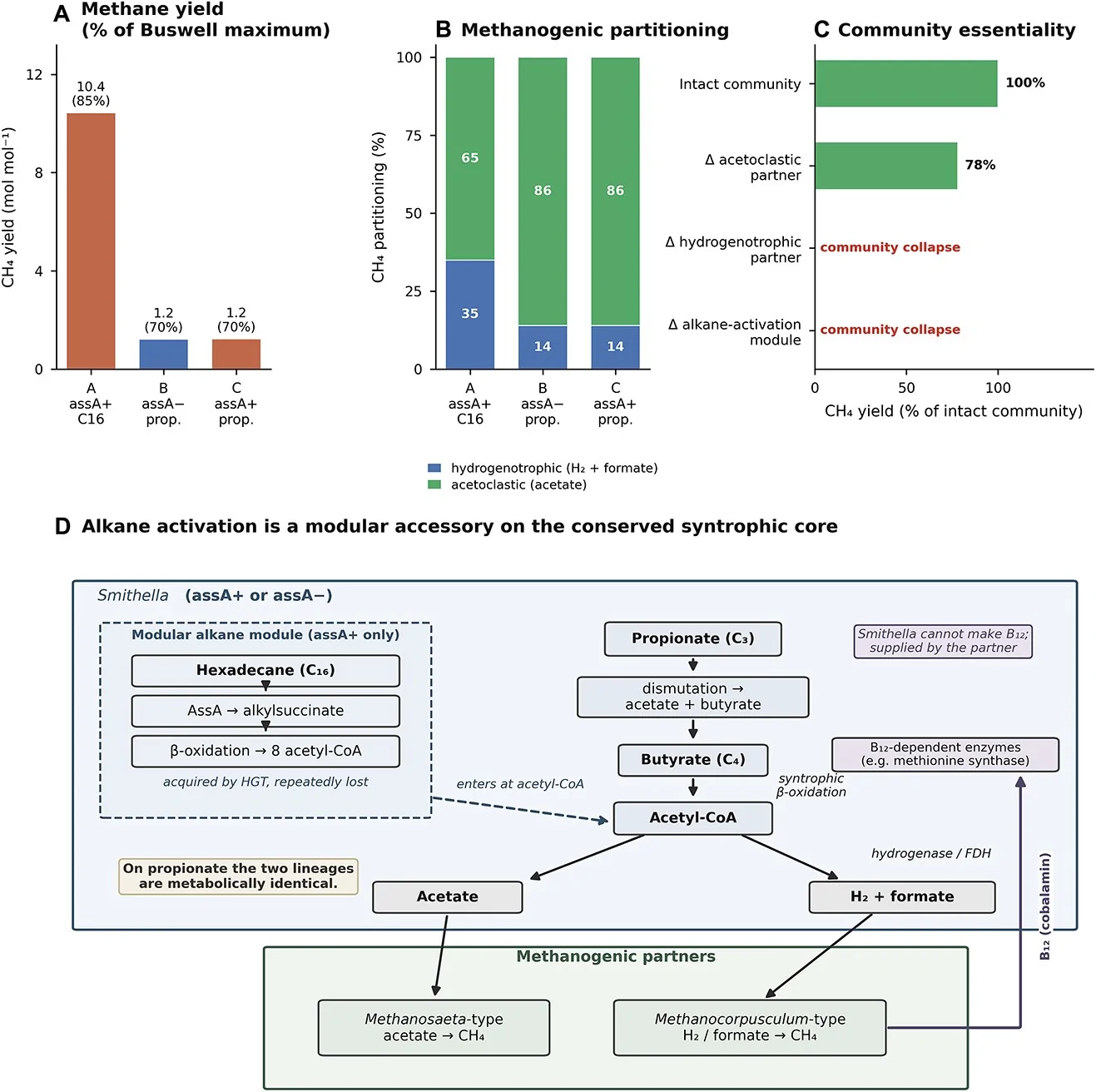
Metabolic modelling of the *assA*+ and *assA*− *Smithella* lineages in a three-member methanogenic consortium. (A) Representative methane yield for three configurations: the *assA*+ lineage on hexadecane (C_1 6_, the canonical *Smithella*-associated *n*-alkane) and both lineages on the shared ancestral substrate, propionate. The higher yield on hexadecane reflects the greater carbon and reductant content of a C_1 6_ alkane, not a metabolic difference between the lineages; the growth-coupled yields are a fixed fraction of the Buswell theoretical maximum (56; 85% on hexadecane, 70% on propionate). The remainder of the substrate enters biomass. (B) Methanogenic partitioning between hydrogenotrophic (H₂ and formate) and acetoclastic (acetate) methanogenesis, set by the degradation stoichiometry (acetoclastic-to-hydrogenotrophic, ~65:35 on hexadecane and 86:14 on propionate). (C) Community essentiality, tested by single-reaction deletions in the *assA*+ hexadecane model (Table S17). Yields are relative to the intact community, whose methane-maximizing yield on hexadecane is 12.25 mol per mol substrate, the Buswell maximum; the growth-coupled yield of panel A is a different quantity, 85% of that. Deleting the alkane-activation module or the hydrogenotrophic partner collapses the community (no growth). Deleting the acetoclastic partner is tolerated, leaving 78% of the intact-community yield, now produced entirely by the hydrogenotrophic route, a stoichiometric consequence of the closed-community design. (D) Schematic of the model. All *Smithella* share a conserved obligate-syntrophic core that oxidizes propionate by dismutation to acetate and butyrate, followed by syntrophic beta-oxidation of the butyrate to acetyl-CoA (the route characterized for the type species; the genomes also encode methylmalonyl-CoA-pathway genes), disposing of electrons as acetate, H₂, and formate, consumed by an acetoclastic (*Methanosaeta*-type) and a hydrogenotrophic (*Methanocorpusculum*-type) methanogen. The alkane-activation module (*assA*+ only; acquired by horizontal transfer, lost independently in several lineages) enters this core at acetyl-CoA, adding a substrate without reorganizing central metabolism. On the shared substrate, the two lineages are therefore metabolically equivalent in the reported outputs. Oxidizing the fully reduced alkane leaves H₂ and formate as the only electron sink, which makes the hydrogenotrophic partner obligatory (Table S17). Cobalamin (B₁₂) is the only *de novo* cofactor pathway lost across the genus. *Smithella* therefore depends on an external corrinoid source, plausibly the methanogenic partner (the anaerobic corrin-ring pathway is incomplete in all 74 *Smithella* genomes while corrinoid salvage is retained; Table S6).

Single-reaction deletions make the stoichiometric basis of the partner requirement explicit. In the *assA*+ model on hexadecane, deleting the alkane-activation module was lethal, as was deleting the hydrogenotrophic partner: a fully reduced alkane oxidized to acetyl-CoA releases reducing equivalents for which H₂ and formate are the only acceptors. Deleting the acetoclastic partner was tolerated, leaving 78% of the intact-community yield with acetate oxidized syntrophically and the electrons passed to the hydrogenotrophic partner (Fig. 5C; Table S17). Partner essentiality is reported for the hexadecane configuration only, where the electron balance closes under the closed-community constraint (Supplementary Methods).

The genomes encode the dismutation route’s butyrate branch in full and only part of the methylmalonyl-CoA pathway, and gene content alone does not indicate which operates. The thermodynamic basis for favouring dismutation was established by Dolfing [56]: it releases one H₂ per propionate against three for the methylmalonyl-CoA pathway, remaining exergonic at higher hydrogen pressures while producing more acetate and being more product-inhibited. The implementation here reproduces Dolfing’s values to within 0.1 kJ (Table S7). At 1 mM acetate, the routes are equienergetic at 4.7 × 10^−5^ atm H₂, within the 10^−5^ to 10^−4^ atm range typical of methanogenic environments. Dismutation is the more exergonic only above that pressure and only below ~8 mM acetate (Fig. S5). Dismutation is therefore favoured where hydrogen removal is imperfect and acetate removal efficient, conditions not measured in the enrichments from which the genus is recovered [14, 15, 18–20].

The genus-scale genomes further address whether *Smithella* retains the alternative route. Methylmalonyl-CoA epimerase, which interconverts the product of the carboxyltransferase and the substrate of the mutase, is absent from all 18 *assA*+ genomes and from 79% of *assA*− genomes. The same orthogroups are recovered in 81% of UBA8904, 62% of MVRV01, and in the *Syntrophus* outgroup. The absence therefore satisfies the between-genus positive control (Table S7). Two cautions bound this. No orthogroup in the 109-genome catalogue is annotated as the committed B₁₂-dependent methylmalonyl-CoA mutase (the *ass*-island *hcmAB* is a different mutase). That step has no positive control and is not counted as a loss. The enzymes catalysing the dismutating step itself have never been identified [11, 64]. Although the butyrate β-oxidation branch is complete in every *Smithella* genome, that is consistent with, but not evidence for, the route.

Beyond electron disposal, the reconstructions indicated a second, orthogonal dependence on the methanogenic partner. Because gap-filling can add reactions absent from the genome, we screened by profile-HMM for the committed enzyme of every cofactor implicated in syntrophic cross-feeding (Table S6). Ten of 12 are synthesized *de novo*, their committed enzymes present in 86%–96% of genomes. Menaquinone alone is unresolved: *menB* is detected in about a quarter of genomes in each genus, and in the outgroup (Table 2 by orthogroup; Tables S10 and S5B by profile-HMM). Loss cannot be separated from incompleteness. The exception is cobalamin, where the whole pathway is lost: CbiA and the ring-assembly enzymes CbiD, CbiH, CbiJ, and CbiL are each detected in at most one of the 74 genomes, while MVRV01 retains them in 43%–86% of its high-quality genomes. Corrinoid salvage and adenosylation are retained (CobO 97%, CobT 41%, CobS 34%) alongside a transporter (BtuF 84%), consistent with uptake and remodelling of a corrinoid *Smithella* cannot build [9, 65]. Cobalamin is the only *de novo* cofactor pathway lost across the genus.

### Physical attachment and interspecies electron transfer

All *Smithellaceae* genomes encode a broadly conserved Type IV pilus (T4P) assembly system, with *pilQ*, *pilB*, *pilC*, and *pilT* each in at least 92% of genomes and *pilQ* in every *Smithella* genome (Table S16), consistent with a role for cell–cell attachment in syntrophic growth. Genuine Type IVa PilA were scored on mature-peptide aromatic-residue density and largest aromatic-free gap, calibrated against three conductive positive and two negative controls (Methods; Fig. 6A; Table S3). Most *Smithella* PilA do not reach the conductivity thresholds: only a minority meet the aromatic-density threshold under the permissive Walker *et al.* [48] filter (*assA*+ 2 of 5, *assA*− 6 of 25 genuine pilins), and almost none meet the stricter Bray *et al.* thresholds, which even the conductive outgroup *S. aciditrophicus* SB (including its validated pilin SYN_00814) satisfies only permissively. A conductive-pilus capability, and any difference between the lineages, is therefore advanced only as a hypothesis.

**Figure 6:**
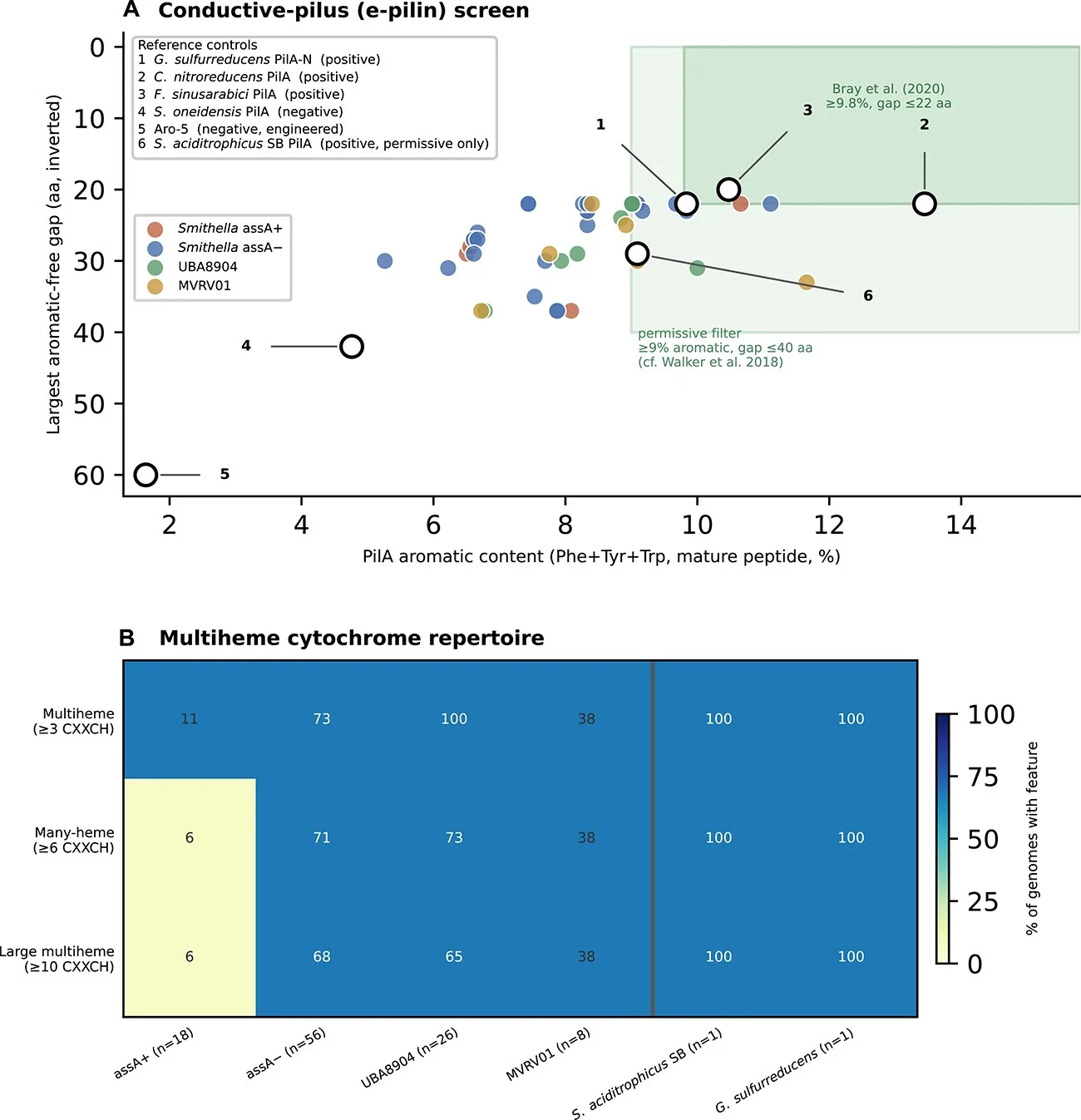
Candidate DIET potential across the *Smithellaceae* (unconfirmed hypothesis). Two independent, sequence-based screens of DIET capability, with experimentally characterized reference organisms run through the same pipelines for calibration. (A) Conductive-pilus (e-pilin) screen. Type IVa PilA major pilins are plotted by mature-peptide aromatic-residue density (phenylalanine, tyrosine, and tryptophan; *x*-axis) and largest aromatic-free gap (*y*-axis, inverted so that stronger candidates lie towards the upper right); colour denotes lineage. The shaded regions mark a permissive filter applying the criteria of Walker *et al.* [48] and the stricter, nested thresholds of Bray *et al.* [50]; both threshold pairs are given in the Supplementary Methods. Numbered points are reference pilins scored by the same screen: three experimentally conductive positives (1, *G. sulfurreducens* PilA-N; 2, *Calditerrivibrio nitroreducens* PilA; 3, *Flexistipes sinusarabici* PilA), two negatives (4, the low-aromatic *Shewanella oneidensis* PilA; 5, the engineered Aro-5 pilin), and the *S. aciditrophicus* SB pilin SYN_00814 [6]. The three reference positives fall at or inside the zones and the negatives well outside; the screen therefore separates the characterized extremes, but its sensitivity is limited, as the conductive *Syntrophus* pilin below reaches only the permissive zone. (B) DIET-associated cytochrome repertoire, scored per genome as three complementary markers of increasing haem-richness: multiheme, many-heme, and large multiheme c-type cytochromes (at least 3, 6, and 10 CXXCH motifs, respectively). For the four *Smithellaceae* lineages, the cell value is the percentage of genomes in that MAG pool carrying the feature (pool size n on the axis). The two right-hand columns are single experimentally conductive reference isolates (the outgroup *S. aciditrophicus* SB and *G. sulfurreducens*; *n* = 1 each). For those two, the value denotes presence (100) or absence (0) in that one genome; it is not a population frequency. Both score across all tiers, but the screen indexes the presence of multiheme-cytochrome genes, not their abundance or their use in DIET. SB is a positive control for the pilin route only (49; panel A), not a cytochrome-based one, encoding ~6 multiheme cytochromes against roughly 70 in *Geobacter* (Table S18). The pattern is nonetheless presented only as a hypothesis, because *assA*+ genomes are fewer and less contiguous and DIET is not experimentally tested here. Full per-pilin and per-genome results are in Tables S3, S4, and S18.

The classical H₂/formate-based indirect electron-transfer system is near-universally conserved: [FeFe] hydrogenases in 73 of 74 *Smithella* genomes, including 17 of 18 *assA*+, and formate dehydrogenase in 80%–92% (Table 2). The Hdr/Mvh complex proposed by Qin *et al.* [14] as an alternative ferredoxin-regenerating system in Ca. *S. cisternae* M82_1 is conserved across the genus (89%, both lineages; Table 2), supplementing rather than replacing the core IET machinery.

A second, independent indicator of DIET potential, the genome’s content of DIET-associated cytochromes, also places the *assA*+ lineage at the low end of the family distribution (Fig. 6B). Across three tiers of increasing haem-richness (Fig. 6B), *assA*+ prevalence is 11%, 6%, and 6%, against 73%, 71%, and 68% in *assA*− and 100%, 73%, and 65% in UBA8904. *Geobacter sulfurreducens* scores at all three tiers and validates the survey; *S. aciditrophicus* SB does too, but conducts DIET through pili [49] and carries far fewer multiheme cytochromes (~6 against 70; Table S18). It controls the pilin screen only (Fig. 6A). The ranking is a hypothesis: *assA*+ genomes are fewer (18 versus 56) and less contiguous (median contig N50 ~14 versus 32 kb), and multiheme cytochromes are fragmentation-sensitive. The difference can be neither confirmed nor excluded (Fig. 6).

## Discussion

*Smithella* has been characterized largely through individual genomes from single enrichment cultures [11, 14, 15, 26, 27], which established its two signature capabilities, propionate dismutation and methanogenic alkane activation, but left open how the genus is organized and how these capabilities are distributed within it. Analysing 74 *Smithella* genomes in a genus-scale framework answers this: the genus is a diverse but coherent obligately syntrophic lineage built on a conserved propionate-oxidizing core, onto which alkane activation is grafted as a horizontally acquired accessory function lost independently in several lineages, on a background of progressive genome reduction.

### Energy limitation and partner dependence

As a check on the gene-content reconstruction, the consortium models recover the partner requirement established in culture [15]. The partner requirement follows from the electron sink: alkane oxidation leaves only H₂ and formate as acceptors, whereas propionate dismutation would retain part of the reductant in butyrate carbon. That route’s epimerase is absent from all *assA*+ and most *assA*− genomes, though the sister genera largely retain it (Table S7); its recovery in equally complete sister genomes makes genuine loss plausible, which would leave those lineages dependent on dismutation where hydrogen accumulates. Partner dependence is not limited to electron disposal: *Smithella* exports reduced one- and two-carbon products and depends on the partner both to keep oxidation feasible and to supply the cobalamin it can no longer synthesize.

### Progressive genome reduction

That dependence is one consequence of a broader reductive trajectory. Ancestral-state reconstruction across three methods indicates pervasive reduction, paralleling genome streamlining in other energy-limited symbiotic or syntrophic systems [66, 67], as expected under chronic energy limitation. *Desulfatibacillum alkenivorans* AK-01 shows this is not a consequence of alkane degradation: it degrades alkanes via fumarate addition yet retains sulfate reduction, flagellar motility, and broad metabolic versatility in a 6.5 Mb genome [68].

The individual losses describe a progressive, cumulative commitment to syntrophy: flagellar deletion at the *Smithella* stem, loss of the dissimilatory sulfite reductase, loss of *de novo* cobalamin biosynthesis creating dependence on an external corrinoid source, and acquisition of the *ass* operon by horizontal transfer onto the retained propionate-oxidizing core (Table 2 and Fig. 3). The three losses all satisfy the between-genus positive control applied throughout; menaquinone does not, and is reported as unresolved.

### Direct interspecies electron transfer remains unresolved

Whether *Smithella* also retains a direct electron-transfer route remains open: the close relative *S. aciditrophicus* makes conductive pili and can perform DIET [49], and DIET has been shown between *Geobacter* and *Methanosarcina* [69], though some apparently conductive pairings in fact exchange hydrogen and formate [70]. Both e-pili and cytochrome markers form a family-wide gradient lowest in the *assA*+ lineage (Fig. 6), but *Geobacter*-calibrated thresholds are uncertain outside that group and *assA*+ genomes are few and fragmented. This remains a hypothesis.

### Implications for subsurface carbon cycling and functional annotation

These results indicate that *Smithella* should not be treated as a single metabolic guild. Its diversity survives the completeness filter. Gene content is organized by species, and each *assA* group is a genomically diverse assemblage differing mainly in substrate range. Alkane degradation is distributed across several species, and community function may depend on which are present. These are genomic and modelling inferences without functional proof; 48% of the 9913 orthogroups encode proteins of unknown function.

The close metabolic coupling with methanogenic partners raises the possibility of gene exchange across the domain boundary. Screening the proteomes for archaeal-origin genes supported none, including *assA*, whose relatives are bacterial. The candidates returned came from methanogen-derived contigs in one assembly dropped by r232 quality control (GCA_964400695.1), where the genes are ~96% identical to a co-occurring *Methanocorpusculum* but only ~40% identical to their *Smithellaceae* orthologs, indicating misbinned contamination rather than transfer (Tables S19 and S20).

Our analysis places previous single-genome and enrichment-based observations [14, 15, 71] in a genus-scale framework: *assA*+ genomes are distributed across multiple species-level lineages rather than forming one isolated alkane clade, making independent gains and losses central to the genus’s history. These remain genomic inferences, awaiting functional and cultivation tests in a genus still largely uncultured.

## Supplementary Material

Supplementary_material_ycag275

## Data Availability

All metagenome-assembled genomes analysed in this study are publicly available; individual accession numbers are listed in Table S8. The genome-scale metabolic models (*assA*+ pan-GEM and *assA*− pan-GEM in SBML format), custom analysis scripts, and OrthoFinder orthogroup definitions have been deposited at Zenodo (https://doi.org/10.5281/zenodo.19148249) and are publicly available. The *S. aciditrophicus* SB reference genome is available under accession GCF_000013405.1.
