# Supplementary material for: Comparative genomics of the Smithellaceae: reductive evolution and a mobile alkane-activation module in Smithella
Source: ISME Commun. 2026 Sep 28;6(1):ycag275. doi: 10.1093/ismeco/ycag275 (PMC13685792; doi:10.1093/ismeco/ycag275)
Supplement: Supplementary file 1 [file supplementary_material_ycag275.zip › Smithella_supplementary_V4.docx]

**Supplementary Information**

**Comparative genomics of the *Smithellaceae*: reductive evolution and a mobile alkane-activation module in *Smithella***

**I. SUPPLEMENTARY METHODS**

Summaries of genome selection, phylogenomics, comparative genomics and ancestral state reconstruction are given in the main-text Methods; the procedures below expand them and are the full record for the conductive-pilus and direct interspecies electron transfer (DIET) cytochrome surveys, the archaeal-gene screen and the metabolic modelling.

**SM1. Phylogenomic analysis**

In addition to the GTDB-Tk bac120 (1) phylogeny described in the main text, a second species tree was inferred from OrthoFinder v2.5.5 (2) orthogroups. MAFFT v7.525 (3) aligned 914 single-copy orthogroups shared by 95% or more of genomes, and IQ-TREE2 v2.3.6 (4) inferred a maximum-likelihood tree under the Q.pfam+R4 model with 1000 UFBoot and 1000 SH-aLRT replicates (5). This tree was used for ancestral state reconstruction (Dollo parsimony) (6) because it captures the full set of shared orthogroups rather than a fixed marker set.

*AssA* status was determined by hmmsearch (7) against a curated AssA HMM profile (847 positions), validated by BLASTp against eight published AssA reference sequences (pident ≥50% threshold). Genomes with fragmented HMM hits, due to contig breaks within the *assA* coding region, were additionally screened by tBLASTn of a full-length *Smithella* AssA query (836 aa) against the assembly; three genomes were confirmed as *assA*+ based on high-identity partial coverage (80–87% protein identity across 41–54% of the reference length) despite assembly fragmentation. These three genomes were excluded from the *assA* gene tree owing to insufficient sequence length for reliable phylogenetic placement but are included in all other *assA*+ analyses.

Ancestral state reconstruction. Orthogroup presence/absence was reconstructed across the species tree by Dollo parsimony over the 9,902 orthogroups that vary within the 109-genome set. As a sensitivity analysis the same states were reconstructed in castor under a two-state Mk model and by maximum parsimony, and the ancestral calls at the *Smithella* most recent common ancestor were compared between methods. For *assA* specifically, equal-rates and asymmetric two-state models were fitted in ape (28) and compared by likelihood-ratio test, and 500 stochastic character maps were then generated under the asymmetric model in phytools (29) with a fixed seed; the gain and loss counts and the posterior probability of a single gain quoted in the main text are taken from that mapping.

Species delimitation and terminal-branch losses were both checked for sensitivity. Single linkage at 95.5% ANI is chain-prone. The same pairwise skani matrix was therefore re-clustered under average and complete linkage. Both give 24 species among the 74 *Smithella* genomes against 23 under single linkage, with the same largest cluster (21 genomes) and the same five species carrying *assA*. The species-level claims therefore do not depend on the linkage rule. Terminal-branch loss counts scale with genome completeness (mean 561 over all *Smithella* tips, 504 above 90% completeness, 461 above 95% and 404 above 99%; regression to complete genomes gives 469). The reported mean is an upper bound inflated by roughly a fifth rather than by several fold.

**SM2. Comparative genomics pipeline**

Functional characterisation of unannotated orthogroups. A total of 283 orthogroups lacking functional annotation from the initial Prokka (9) /eggNOG-mapper (10) pipeline were characterised by three complementary approaches: InterProScan database queries (Pfam, TIGRFAM, PANTHER, CDD, SUPERFAMILY, Gene3D, PRINTS, ProSiteProfiles, HAMAP), genomic context analysis (±5-gene neighbourhood), and codon usage/GC content analysis to flag HGT candidates. Orthogroups with no detectable sequence homology (n = 114) were subjected to structure-based homology search using ESMFold (11), with AlphaFold2 for the cases noted in Table S2, and Foldseek (12) against PDB100 (91/114 structures predicted; matches classified by probability ≥0.9 confident, 0.5–0.9 possible). For the dark proteome, 100 representative sequences from the most conserved unannotated orthogroups were further analysed using ColabFold v1.5 (13) implementing AlphaFold2 (14); 89/100 returned significant structural hits (TM-score >0.5), with the majority encoding housekeeping functions. This pipeline also exposed cascading Prokka misannotations. OG0002445 encodes clpP_2 (~260 aa) with no GRE motif, and OG0002508 - auto-annotated *bssA* and used here as the *assA* presence/absence marker - is a ~123 aa protein encoded immediately upstream of *assA*, not the catalytic subunit. Alignment against the *Desulfatibacillum* AK-01 *ass* operon identifies it as the alkylsuccinate synthase beta subunit (Dalk_1729, 122 aa; 38% of self-score), and OG0002507, auto-annotated as the *bssD* activase, as the gamma subunit (Dalk_1730, 54 aa; 61%). Both eggNOG assignments are therefore misassignments, and the two orthogroups are subunits of the holoenzyme rather than neighbouring genes. The catalytic *assA* CDS (~836 aa) is not resolved as its own orthogroup: its members fall in OG0000023, a lumped glycyl-radical-enzyme orthogroup auto-annotated *pflD* and spanning 101 genomes. OG0002508 is nonetheless present in 17 of 18 *assA*+ and 1 of 56 *assA*- genomes and therefore tracks *assA* closely; the *assA* calls used throughout come from the curated AssA HMM and tBLASTn (SM1; per-genome calls in Table S8), not from orthogroup membership.

Flanking-synteny comparison. Genes within +/-20 kb of each *assA* copy were compared by orthogroup Jaccard similarity, restricted to the 19 of 23 loci with at least five flanking genes recovered (the remainder are contig-edge fragments). Because pairwise similarities share loci and are not independent, groups were compared by exact permutation over locus labels rather than by a rank test over pairs: all C(19,8) = 75,582 label assignments were enumerated, giving p = 5.3 × 10⁻⁵ for the within-A versus within-B contrast. The two *Desulfatibacillum alkenivorans* AK-01 ass loci (CP001322.1; the Dalk_1728-1731 and Dalk_2198-2202 operons taken with their flanking context, 31 and 38 CDS) and the *Azoarcus* sp. HxN1 mas operon (AM748709.1, masA-masG, 7 CDS) were retrieved from GenBank and their genes assigned to *Smithella* orthogroups by local alignment at a threshold of 30% of self-score; of 76 reference CDS, 30 scored above 0.30 and only 3 fell between 0.15 and 0.30. Median contig N50 is 13.4 kb across the family A host genomes and 20.8 kb across the family B host genomes (Mann-Whitney p = 0.44). Family A carries the lower contiguity and the higher flanking-gene similarity; the two run in opposite directions. The ±20 kb island averages 3.3 percentage points more G+C than its host genome, and the *assA* gene about 8 percentage points. The exact permutation test is implemented in analysis/permutation_test_synteny.py of the deposited archive.

**SM3. Metabolic modeling procedures**

This supplement provides the technical details needed to support the comparative *Smithellaceae* modelling in Fig. 5 and Table 1. The aim of the model is to test whether the *assA*+ and *assA*− *Smithella* lineages differ in their core syntrophic metabolism, or whether alkane degradation is a modular addition to a shared core.

**Model construction**

The two central models are lineage-level pan-genome-scale metabolic models for the *assA*+ and *assA*− *Smithella* lineages, reconstructed with gapseq v1.4.0 from the GTDB-r232-validated representative genomes. Each model was gap-filled against a minimal anaerobic medium with its substrate as the sole carbon source and no vitamins or cofactors supplied, so that it is a self-sufficient anaerobe that synthesises its modelled biomass precursors de novo. This self-sufficiency is a property of gap-filling and does not bear on the gene-level cobalamin auxotrophy identified by the comparative genomics (see below). Only the alkane-activation module was added to the *assA*+ model, as two mass- and charge-balanced reactions: AssA-mediated fumarate addition (hexadecane + fumarate to (1-methylpentadecyl)succinate) and oxidation of the C16 chain to eight acetyl-CoA with fumarate regenerated. The electron yield of the oxidation is fixed by the degree of reduction (34 electron-equivalents, 17 [2H], carried as NADH) rather than assigned by hand. No other lineage-specific reactions were introduced. Each *Smithella* model was analysed in a three-member consortium with a hydrogenotrophic *Methanocorpusculum*-type sink (H2 and formate) and an acetoclastic *Methanosaeta*-type sink (acetate); community fluxes were solved with the parameter-free SteadyCom formulation (equal specific growth rate), cross-checked with MICOM v0.37.1 (cooperative-tradeoff fraction 0.45).

Two closed three-member consortia were analysed. In the *assA*− configuration, *Smithella* degraded propionate; in the *assA*+ configuration, *Smithella* degraded hexadecane after AssA-mediated activation. Community export of H2, acetate, and formate was blocked so that syntrophic products had to be consumed internally. The parameter-free SteadyCom formulation was used for the steady-state analyses, cross-checked with MICOM v0.37.1 (15). Flux-variability analysis was applied to the steady-state solutions to identify which community fluxes are uniquely determined; the partitioning of hydrogenotrophic methanogenesis between H2 and formate was found to be non-unique and is therefore not reported (see main text). The acetoclastic-to-hydrogenotrophic split is reported from the substrate's degradation stoichiometry rather than the flux solution, because a stoichiometric community model reproduces the total methane but cannot forbid the thermodynamically unfavourable syntrophic oxidation of acetate. Knockout results are reported for the hexadecane configuration only. In the propionate configurations the community could satisfy its electron balance only by exporting reduced menaquinone, an artefact of the reconstruction rather than a syntrophic route, and with that export closed the propionate community admits no solution. The propionate configuration is therefore used for yield and partitioning only.

The deposited archive contains the two pan-genome-scale models (assA_plus_panModel.xml and assA_minus_panModel.xml), the two minimal partner models (a hydrogenotrophic *Methanocorpusculum*-type sink and an acetoclastic *Methanosaeta*-type sink), and the scripts that build the three-member community, run the SteadyCom analysis and reproduce the reported methane yields, partitioning and knockout tests. Software versions are recorded with the deposit.

Cross-check with MICOM. The community results reported here use the parameter-free SteadyCom formulation (24), which imposes a single shared specific growth rate across all members and carries no tradeoff parameter. Re-analysing the same models with the MICOM cooperative-tradeoff objective gave the same qualitative outcome: both lineages self-sufficient obligate syntrophs, equivalent reported outputs on propionate, and acetoclastic-dominant methanogenesis, confirming that the conclusions are not specific to the community-FBA method. Both methods reach the Buswell methane maximum on hexadecane when methane is maximised (12.25 mol CH4 per mol); on propionate the intact community reaches 1.71 against a Buswell maximum of 1.75; the reported acetoclastic-to-hydrogenotrophic split (about 65:35 on hexadecane, and about 86:14 on propionate, dismutation route) is taken from the degradation stoichiometry, since neither stoichiometric method constrains it.

We report the propionate partitioning from the dismutation route (propionate to acetate and butyrate, then syntrophic beta-oxidation of the butyrate) characterised for the type species *Smithella* *propionica* by 13C labelling (25), which gives a strongly acetoclastic split (about 86:14) and is the feature distinguishing *Smithella* from other propionate-oxidising syntrophs. The pan-GEM additionally encodes methylmalonyl-CoA-pathway genes (which would give about 57:43), but gene presence does not establish the operative pathway; the total methane is identical for both routes. We report the split for the characterised route and flag the encoded methylmalonyl-CoA genes as an unresolved gene-versus-biochemistry tension.

**SM4. Genomic feature surveys**

***Flagellar gene survey and sulfite reductase loss***

Zero flagellar structural genes were detected in any of 74 *Smithella* genomes. Over 30 flagellar-associated orthogroups (*fliC, flgB through flgL, flhA/B, motA/B*) are present in the sister genera of the *Smithellaceae* but entirely absent from the *Smithella* lineage, consistent with loss of motility. Chemotaxis signalling genes (*cheA*, *cheW*, *cheY*) are retained in 78 to 100% of *Smithella* genomes, likely repurposed for pilus-based taxis or biofilm regulation. In contrast, flagellar genes are retained in UBA8904 (10 to 13 of 26 genomes, 38 to 50%) and MVRV01 (8 of 8 genomes, 100%), indicating that flagellar loss is specific to the *Smithella* clade (Table S16 in Supplementary_Tables_REVISED.xlsx).

Dissimilatory sulfite reductase (*dsrAB*) is absent from all 55 high-quality *Smithella* genomes by profile-HMM (TIGR02064/TIGR02066, hmmsearch --cut_ga), while the sister genera retain it (UBA8904 53%, MVRV01 29% of high-quality genomes), providing the between-genus positive control for the loss. The sulfate-activating enzymes *sat* and *aprAB* are absent from every *Smithellaceae* genome. No genome encodes a complete dissimilatory sulfate-reduction pathway; the loss in *Smithella* is specifically of the dissimilatory sulfite reductase.

**Conductive-pilus (e-pilin) and DIET-cytochrome surveys**

Conductive-pilus (e-pilin) potential (20) was screened from the PilA major pilin in two steps. Candidate pilins were first gated for genuine type IVa identity (a class III prepilin signal peptide with the PilD G–F cleavage motif, the conserved N-terminal hydrophobic α-helix and the near-invariant Glu5), and only genuine pilins were scored. On the mature peptide the aromatic-residue density (phenylalanine, tyrosine and tryptophan) and the largest aromatic-free gap were computed, and a permissive filter approximating the qualitative criteria of Walker et al. (21) (density ≥9%, largest aromatic-free gap ≤40 residues) was applied together with the stricter numeric thresholds of Bray et al. (22) (density ≥9.8%, gap ≤22 residues); aromatics at the conserved *Geobacter* positions (residues 24, 27, 32, 51 and 57) were recorded as corroborating, alignment-dependent evidence only. The screen was calibrated with reference pilins run through the same pipeline: three experimentally conductive positives (*Geobacter sulfurreducens* PilA-N, *Calditerrivibrio nitroreducens* PilA and *Flexistipes sinusarabici* PilA) and two negatives (the low-aromatic *Shewanella oneidensis* PilA and the engineered Aro-5 pilin). Full per-pilin results are in Table S3.

DIET-associated cytochrome content was surveyed as a holistic per-genome repertoire at increasing haem-richness: multiheme c-type cytochromes (≥3 CXXCH motifs), many-heme cytochromes (≥6 CXXCH motifs) and large multiheme cytochromes (≥10 CXXCH motifs). *Geobacter* *sulfurreducens* is the cytochrome-rich reference, and the outgroup *Syntrophus* *aciditrophicus* SB, which conducts DIET through conductive pili (23), is the second reference organism. The screen scores the presence of multiheme-cytochrome genes rather than their abundance or their use in DIET. Full per-genome results are in Table S4.

**SM5. HGT detection and validation**

Methods for the archaeal-affiliated gene screen are described below. Evaluated over the quality-controlled r232 genome set, no candidate is supported as an archaeal transfer. The apparent signal is attributable to methanogen-derived contigs carried in GCA_964400695.1, a 61.2%-complete assembly not included in the quality-controlled genome set. One example is a *larB* copy on the 3,986 bp contig CAYNVU010000303.1, about 96% identical to *Methanocorpusculum* and about 40% identical to its *Smithella* orthologs. The candidate list and the per-orthogroup assessment are given in Tables S19 and S20.

Archaeal-origin genes were detected by two complementary approaches: the Alien Index (16), calculated as AI = (best bacterial bitscore − best archaeal bitscore) / (best bacterial bitscore + best archaeal bitscore), and taxonomic distribution of the top 20 DIAMOND (17) BLASTp hits against NCBI nr (707M sequences). A two-method consensus flagged 64 orthogroups, of which 20 recurred across at least two *Smithellaceae* genera and are listed in Table S19. Fifteen of those 20 had enough nr references for phylogenetic assessment (the remaining five are marked “not testable” in Table S20); it used IQ-TREE2 gene trees with multiple *Smithellaceae* representatives and long-branch-attraction controls (MAFFT alignment (3), trimAl trimming (18), ModelFinder (19) model selection, 1,000 UFBoot replicates (5), rooted on bacterial outgroups). MAG contamination was assessed by examining contig context: candidates on contigs with ≥3 bacterial-flanking genes and present in ≥3 genomes across ≥2 genera were classified as candidate HGT (Table S20).

**SM6. Thermodynamics of the two propionate-oxidation routes**

The two propionate-oxidation routes encoded in the *Smithella* genomes were compared thermodynamically, following the treatment of Dolfing (26), whose standard free-energy values for the two routes this implementation reproduces. Standard Gibbs free energies of formation (27) were used to obtain the free-energy change of each route at pH 7. The actual change was then evaluated across the physiological ranges of hydrogen partial pressure (10⁻⁷ to 10⁻¹ atm) and acetate concentration (0.1 to 10 mM) at 1 mM propionate and 10 mM bicarbonate. The implementation returns +48.4 kJ per 2 propionate for dismutation and +76.4 kJ per propionate for the methylmalonyl-CoA route, reproducing Dolfing's +48.4 and +76.5 to within 0.1 kJ/mol. (Both reactions are written with bicarbonate; the +48.1 and +76.1 values quoted in earlier drafts are Thauer et al. (27) values for the CO2 form, and the script computes neither butyrate oxidation nor those values.) This is a stoichiometric and thermodynamic calculation independent of the genome-scale models; it is implemented in analysis/propionate_route_thermodynamics.py of the deposited archive, which also draws Figure S5.

II. **SUPPLEMENTARY RESULTS**

**SR1. Phylogenomic framework**

Figure S1 shows pairwise average nucleotide identity (ANI) among the 74 *Smithella* genomes (skani, GTDB r232), grouped into the 23 species resolved by single-linkage clustering at 95.5% ANI. *assA* is present in only 5 of the 23 species, and the *assA*-containing clusters are interspersed among *assA*-negative ones rather than grouped together; one species contains both *assA*+ and *assA*- genomes. This distribution is consistent with horizontal redistribution and repeated loss of the *assA* island rather than a single vertical loss event. Fifteen of the 23 species contain no genome carrying an assigned GTDB species, indicating diversity beyond the currently named taxa. The ANI framework underpins the species-level analyses used throughout this study.

**SR2. Comparative genomics and evolutionary patterns**

Pangenome partitioning across lineages (Figure S2) reveals that *Smithella* has a smaller core genome and proportionally more lineage-specific genes than UBA8904 or MVRV01, consistent with greater niche specialisation. Functional enrichment analysis between the *assA*+ and *assA*- clades highlights the alkane-degradation island as the primary feature that differentiates the two groups.

To confirm that *assA* absence in *assA*-negative clades reflects genuine gene loss rather than assembly fragmentation, we performed tBLASTn of a full-length *Smithella* AssA query (836 aa, from GCA_030018675.1) against all 74 *Smithella* genomes; a within-genus query is the more sensitive test for residual *assA*. Figure S3 shows that no *assA*-negative genome retains a full-length *assA*: in each of the 56 genomes the best tBLASTn hit is a partial match to a related glycyl-radical enzyme at 24 to 34% identity, and the distribution of best-hit identities is bimodal with no intermediate values.

**SR3. Metabolic modeling results**

Both models are self-sufficient on their substrate: gap-filled with the substrate as the sole carbon source and no vitamins or cofactors supplied, each satisfies its modelled biomass demands within the gap-filled network. This cofactor self-sufficiency is a property of the gap-filled flux model and does not contradict the gene-level cobalamin auxotrophy identified by the comparative genomics, which the flux model cannot detect because gap-filling restores the missing biosynthetic steps. Run on the shared substrate, propionate, the two lineages are metabolically near-identical in constraint structure and partitioning. Alkane degradation in the *assA*+ lineage therefore is a modular addition to a shared core rather than a divergence within it.

***Limitations***

The consortium architecture is deliberately minimal and asymmetric: *Smithella* is represented by a genome-scale model, while the partner methanogens are represented by minimal stoichiometric sinks. The model therefore supports comparative statements about *Smithellaceae* constraint structure, but it should not be treated as a realistic ecological model of whole methanogenic communities.

Because H2, acetate, and formate export were blocked, some lethality results reflect the closure design rather than universal ecological requirements. In particular, the dependence on the hydrogenotrophic partner in the closed model should not be interpreted as proof that hydrogenotrophic methanogenesis is always required in nature; the acetoclastic partner is not essential, its removal leaving 78% of the intact-community methane yield.

III. **SUPPLEMENTARY FIGURES**

Figure S1. Pairwise average nucleotide identity (ANI) among the 74 *Smithella* genomes (skani; GTDB r232). Genomes are ordered by species cluster, largest first, and white lines delimit the 23 clusters obtained by single-linkage clustering at 95.5% ANI (column “ANI Species (95.5%, single-linkage)” of Table S8). Each cluster is labelled with its GTDB r232 species and the number of genomes it contains; the fifteen clusters that contain no genome with a GTDB species assignment are labelled “no GTDB species” in red. Asterisks mark the five clusters containing *assA*, and the strip at the left gives *assA* status per genome (orange, *assA*+; grey, *assA*−). Colour scale shows ANI (%); values below skani's reporting limit of about 85% are shown at the 80% floor. Generated by analysis/plot_figS1_ani_heatmap.py of the deposited archive, which regenerates the clustering from the raw skani matrix and asserts agreement with Table S8.

Figure S2. Differential gene content across *Smithellaceae* lineages (GTDB r232; 109 genomes, 9,913 orthogroups). (A) Pan-genome partitioning of orthogroups by lineage (core/widespread, *assA*+-specific, *assA*+-shared, *assA*-specific, UBA8904-specific, MVRV01-specific, and non-*assA*+-shared). (B) Functional categories of orthogroups present in *assA*+ and absent from *assA*- *Smithella*, distinguishing those confined to *assA*+ from those also present in UBA8904 or MVRV01. (C) Functional categories of orthogroups absent from *assA*+ *Smithella*, shown per lineage. (D) Tiered annotation of the *assA*+ *Smithella* dark proteome (hypothetical orthogroups recurrent across the *assA*+ genomes).

Figure S3. Validation of *assA* loss in *assA*-negative *Smithella* (74 *Smithella* genomes, GTDB r232). tBLASTn of a full-length *Smithella* AssA query (836 aa, from GCA_030018675.1) against each *Smithella* genome; a within-genus query is the more sensitive test for residual *assA*. (A) Best tBLASTn hit per genome, plotted as amino-acid identity versus query coverage. The 18 *assA*+ genomes (blue) recover high-identity hits (median 82% identity, 99% coverage; positive control), whereas all 56 *assA*-negative genomes (red) return only low-identity partial hits (24 to 34% identity) to related glycyl-radical enzymes and none fall within the retained-*assA* region (identity at least 60%, coverage at least 80%; shaded). (B) The distribution of best-hit identity is strongly bimodal with no intermediate values, indicating clean deletion rather than gradual pseudogenisation. No *assA*-negative genome retains a full-length *assA* or recognisable island remnant (0 of 56).

Figure S4. Maximum-likelihood phylogeny of glycyl-radical enzymes (GTDB r232). All 23 near-full-length *Smithella* AssA copies, in two paralogue families. All 23 near-full-length AssA proteins recovered from the 15 *Smithella* genomes that carry one (this study) were aligned with reference AssA, BssA, NmsA and PflB sequences (LG+R4 model; 1000 ultrafast bootstrap and 1000 SH-aLRT replicates; rooted on PflB). The *Smithella* copies resolve into two paralogue families, A (red; 9 copies in 9 genomes) and B (blue; 14 copies in 10 genomes), four genomes carrying members of both; multiple copies from one genome are numbered. *Smithella* AssA is paraphyletic rather than a single clade: both AssA copies of D. *alkenivorans* AK-01 (AssA1 and AssA2, the genome's two *ass* loci), *Azoarcus* sp. HxN1 MasD and *Desulfosarcina* sp. BuS5 AssA fall inside the *Smithella* group. Neither *Smithella* family corresponds to a particular AK-01 copy: both are closer to AssA1 (73% and 70% identity) than to AssA2 (70% and 65%). The *Smithella* proteins are therefore a single coherent AssA type rather than several unrelated glycyl-radical enzymes, but they are not resolvably distinct from the AssA of other genera. Filled circles mark nodes with SH-aLRT at least 80 and UFBoot at least 95; open circles mark nodes with SH-aLRT at least 80 or UFBoot at least 80. The number of acquisition events is addressed by the per-family gene-tree versus species-tree tests in the main text.

Figure S5. Free-energy change of the two propionate-oxidation routes encoded in *Smithella* genomes, computed at pH 7 from standard Gibbs free energies of formation. (a) ΔG per mol propionate against hydrogen partial pressure at 1 mM acetate; the shaded band marks the 10⁻⁵ to 10⁻⁴ atm range typical of methanogenic environments. The dismutation route releases one H₂ per propionate against three for the methylmalonyl-CoA route. Its free-energy change is a third as sensitive to hydrogen, and the lines cross at 4.7 × 10⁻⁵ atm, inside that band. (b) Hydrogen partial pressure below which each route is exergonic, as a function of acetate. Because dismutation produces more acetate per propionate, its advantage narrows as acetate accumulates and the two thresholds cross at about 8 mM, above which the methylmalonyl-CoA route is the one that tolerates the higher hydrogen pressure. Values in Table S7.

IV. **SUPPLEMENTARY TABLES**

All supplementary tables are provided in the accompanying file Supplementary_Tables_REVISED.xlsx.

Table S1. Complete orthogroup catalogue for the 109 validated genomes (9,913 orthogroups). Gene names, functional annotations from Prokka, eggNOG-mapper, InterProScan, and Foldseek, plus genome-level presence and absence matrix.

Table S2. Structural annotation of the dark proteome. 100 representative unannotated proteins characterised by AlphaFold2 structure prediction (pLDDT scores) and Foldseek homology search against PDB100 and AlphaFold UniProt50 databases.

Table S3. Conductive-pilus (e-pilin) screen of *Smithellaceae* PilA. For each genuine type IVa major pilin (identity-gated on the class III prepilin signal peptide, the conserved N-terminal hydrophobic helix and Glu5), the mature-peptide aromatic-residue density (Phe+Tyr+Trp), the largest aromatic-free gap, the number of aromatic matches at the conserved *Geobacter* positions, and pass/fail against a permissive filter approximating the qualitative criteria of Walker et al. (2018) and the stricter numeric thresholds of Bray et al. (2020). Reference pilins run through the identical pipeline are included as calibration controls: *the three Syntrophus aciditrophicus* SB pilins, three conductive positives (*Geobacter* *sulfurreducens*, *Calditerrivibrio* nitroreducens, *Flexistipes* sinusarabici) and two negatives (*Shewanella* oneidensis, engineered Aro-5).

Table S4. DIET-associated cytochrome repertoire per genome. For each genome, the number of multiheme c-type cytochromes (at least three CXXCH haem-binding motifs), many-heme cytochromes (at least six CXXCH motifs), and large multiheme cytochromes (at least ten CXXCH motifs), and the maximum haem count in a single protein, with *Geobacter* *sulfurreducens* included as a positive-control reference isolate.

Table S5. Robust gene presence/absence and cofactor-auxotrophy verification. Profile-HMM (TIGRFAM/Pfam) with family-specific trusted cutoffs, restricted to high-quality genomes (at least 90% completeness and at most 5% contamination), validated by a between-genus positive control (sister genera) and an internal positive control; absence is asserted only where validated against the sister genera and surviving all controls. (A) Presence or absence of key features across the four groups (*assA*+, *assA*-, UBA8904, MVRV01) with a verdict. (B) Cofactor-auxotrophy calls per biosynthetic pathway, giving committed-enzyme prevalence in high-quality *Smithella*, all *Smithella* and the sister genera, with a Pfam cross-check. The table is supplied as three worksheets: S5_info (method notes), S5A and S5B.

Table S6. Cofactor auxotrophy screen supporting the interspecies-dependency analysis. For each cofactor implicated in syntrophic cross-feeding, the committed biosynthesis enzyme, its Pfam profile, the number of the 74 *Smithella* genomes encoding it (profile-HMM, trusted cutoffs), and the verdict. Only cobalamin is genuinely absent, and the whole anaerobic corrin-ring pathway is missing rather than a single step; ten of the remaining eleven committed enzymes are present in 86 to 96 per cent of genomes, showing their absence from the annotation-only draft models is an artefact of automated reaction mapping rather than a true metabolic gap. Menaquinone is not counted among them: the Pfam used in this sheet (Chorismate_bind) is not diagnostic, and on the committed enzyme *menB* Tables S10 and S5B return about 31 per cent and call the pathway unresolved.

Table S7. Thermodynamic comparison of the two propionate-oxidation routes. Standard free-energy change at pH 7, hydrogen and acetate released per mol propionate, the free-energy penalty per tenfold rise in hydrogen, and the hydrogen partial pressure below which each route is exergonic across a range of acetate concentrations, with the crossover points. A second block gives the gene content of the two routes across the four lineages. The methylmalonyl-CoA route lacks the epimerase in all *assA*+ and 79% of *assA*- genomes, an absence validated against the sister genera and the outgroup. The butyrate β-oxidation branch is complete in every *Smithella* genome. Because the enzymes of the dismutating step itself have never been identified, that completeness is consistent with the route rather than evidence for it. No methylmalonyl-CoA mutase is annotated in any of the 109 genomes (the ass-island hcmAB is a different mutase). That step has no positive control. Underlies Figure S5.

Table S8. Genome statistics and metadata for 108 *Smithellaceae* MAGs plus the *Syntrophus* *aciditrophicus* outgroup. NCBI accession, GTDB taxonomy (genus and species), ANI-based clade assignment, the species assignment used throughout this work, *assA* status, CheckM2 completeness and contamination, genome size, contig N50, GC content, CDS count, number of contigs, country of origin, geographic location, isolation source, and BioSample/BioProject identifiers. Two cluster columns are given and they are not interchangeable: “ANI Clade” is populated for all 109 genomes and is what Fig. 1 ring H counts; “ANI Species (95.5%, single-linkage)” is defined for the 74 *Smithella* genomes only, is the species assignment quoted throughout the text and labels the Fig. 4 synteny tracks, and is the one to use when checking any per-species count. An empty GTDB species field means the genome carries no species assignment in GTDB r232; fifteen of the 23 species consist entirely of such genomes.

Table S9. Species-unique orthogroups. The 2,741 orthogroups present in only one high-quality *Smithella* species (16 species), with gene name, product, and functional category. 74% are hypothetical/unknown and only ~10% are assignable to metabolism, indicating that species-level diversity resides largely in uncharacterised accessory genes.

Table S10. Cofactor biosynthesis pathway completeness across *Smithellaceae* lineages. Pathway coverage for cobalamin de novo synthesis and salvage, siroheme, haem, folate, vitamin B6 and menaquinone, scored for high-quality *Smithella* against the sister genera.

Table S11. Complete set of orthogroups differentially prevalent between *assA*+ and *assA*− *Smithella*. All 292 orthogroups whose prevalence differs significantly between the 18 *assA*+ and 56 *assA*− genomes (Fisher’s exact test, Bonferroni-corrected p < 0.05); 204 are more prevalent in *assA*+ and 88 in *assA*−. Columns give the gene and product annotation, presence counts and percentages in each lineage, the direction of enrichment, and the raw Fisher p-value. The *assA*-island neighbourhood subset is listed in Table S15.

Table S12. AssA gene-tree versus species-tree congruence (approximately-unbiased test). Log-likelihoods and approximately-unbiased test p-values for the maximum-likelihood AssA protein tree and the bac120 species topology evaluated on the full-length AssA alignment (15 genomes). The species topology is rejected (p-AU = 1.1 × 10⁻⁴⁵; ΔlogL 725), indicating that the AssA gene tree is incongruent with vertical inheritance. The table also gives the Robinson-Foulds comparison of the two topologies (12 nontrivial splits each, 3 shared, RF 18 of a possible 24), and notes that 5 of the 15 AssA sequences are identical. On the 10 distinct sequences RF is 8 of 14. This pooled analysis mixes the two AssA paralogue families and is retained only for completeness. The primary test is now run separately within each family, which removes the paralogy confound and rejects the species topology in both (family A p-AU = 1.3 × 10⁻³, ΔlogL 16.0; family B p-AU <= 2.1 × 10⁻⁵, ΔlogL 175–311 across all twelve combinations of one representative copy per genome; per-combination outputs are included in the deposit). An earlier family B result computed on 7 taxa was withdrawn: it had excluded every genome carrying more than one family B copy.

Table S13. Mobile genetic element enrichment around *assA*. Annotation-independent Pfam-HMM detection (163 mobility-focused profiles, trusted cutoffs) of MGE-related proteins per *assA*+ genome, with the observed versus expected MGE count in a plus/minus 20 kb window around *assA* and the per-genome Poisson enrichment p-value. The test is anchored on the co-located beta subunit and so covers the 17 *assA*+ genomes in which that anchor was recovered. Pooled across those 17 genomes: 4 observed versus 4.1 expected (p = 0.58), indicating no enrichment of mobile elements at the locus. One genome (GCA_030018675.1) is nominally enriched (2 observed, p = 0.012); the assemblies are fragmented and insertion sequences frequently terminate contigs. This test has limited power to exclude a resident element.

Table S14. Ancestral-state reconstruction of propionate-metabolism genes versus *assA*. Per-genus prevalence (*Syntrophus*, UBA8904, MVRV01, *Smithella*) and the state reconstructed at the *Smithella* most recent common ancestor for methylmalonyl-CoA decarboxylase (*mmdA*), pyruvate:ferredoxin oxidoreductase (PorA), acetate kinase (*ackA*) and alkylsuccinate synthase (*assA*). *mmdA* and PorA are reconstructed as present (concordant across maximum-likelihood, maximum-parsimony and the outgroup criterion) and *assA* as absent; *ackA* is ambiguous.

Table S15. Genes in the *assA* genomic-island neighbourhood. The 22 orthogroups in this neighbourhood are listed for genomic context. Fisher's exact p-values are given raw and Bonferroni-corrected for the 7,973 orthogroups tested within *Smithella*; only *uspA2* (OG0003449) is significant after correction. The genome-wide significant set is Table S11.

Table S16. Motility, chemotaxis, and Type IV pilus gene prevalence across *Smithellaceae* lineages. Gene counts per lineage group (*assA*+ *Smithella*, *assA*- *Smithella*, UBA8904, MVRV01). A gene is scored present where any of its paralogous orthogroups is detected; the contributing orthogroups are listed in the final column.

Table S17. FBA knockout essentiality analysis for three configurations: *assA*+ on hexadecane, and both lineages on propionate. Community outcome and interpretation for each single-reaction knockout: on hexadecane, deletion of the alkane-activation module or of the hydrogenotrophic partner collapses the community, whereas deletion of the acetoclastic partner is tolerated and leaves 78% of the stoichiometric reference. Knockouts are reported for the hexadecane configuration only, where the electron balance closes under the closed-community constraint.

Table S18. Multiheme-cytochrome evidence for the two reference isolates. Per-protein CXXCH motif counts and RefSeq identifiers for the multiheme c-type cytochromes of *Syntrophus* *aciditrophicus* SB and *Geobacter* *sulfurreducens*, with the per-genome totals used in Fig. 6b. SB encodes about six multiheme cytochromes against roughly seventy in G. *sulfurreducens*. It is therefore a positive control for the pilin route (Fig. 6a) rather than for a cytochrome-based mechanism.

Table S19. Candidate archaeal-affiliated orthogroups screened in *Smithellaceae*. Alien Index, archaeal-versus-bacterial hit ratio, genome distribution across genera, contig-context flanking, and the best archaeal identity computed both from the longest orthogroup member and from members residing in the quality-controlled genomes, with the per-orthogroup assessment. On the quality-controlled genomes none of the candidates is supported as an archaeal transfer (see table footnote).

Table S20. Gene-tree assessment of the candidate archaeal-affiliated orthogroups on the quality-controlled genome set: per-orthogroup archaeal and bacterial homologue counts, best archaeal identity, *Smithellaceae* monophyly, stem-to-crown ratio, and approximately-unbiased tests of an archaeal-nesting versus a bacterial-inheritance topology. No candidate supports an archaeal origin; the flagship genes branch with bacteria of *Smithella*'s own phylum.

**References**

1.	Chaumeil PA, Mussig AJ, Hugenholtz P, Parks DH. GTDB-Tk v2: memory friendly classification with the genome taxonomy database. Borgwardt K, editor. Bioinformatics. 2022 Oct;38(23):5315–6. doi:10.1093/bioinformatics/btac672

2.	Emms DM, Kelly S. OrthoFinder: phylogenetic orthology inference for comparative genomics. Genome Biol. 2019 Nov;20(1). doi:10.1186/s13059-019-1832-y

3.	Katoh K, Standley DM. MAFFT Multiple Sequence Alignment Software Version 7: Improvements in Performance and Usability. Mol Biol Evol. 2013;30(4):772–80. doi:10.1093/molbev/mst010

4.	Minh BQ, Schmidt HA, Chernomor O, Schrempf D, Woodhams MD, von Haeseler A, et al. IQ-TREE 2: New Models and Efficient Methods for Phylogenetic Inference in the Genomic Era. Teeling E, editor. Mol Biol Evol. 2020 Feb;37(5):1530–4. doi:10.1093/molbev/msaa015

5.	Hoang DT, Chernomor O, von Haeseler A, Minh BQ, Vinh LS. UFBoot2: Improving the Ultrafast Bootstrap Approximation. Mol Biol Evol. 2018;35(2):518–22. doi:10.1093/molbev/msx281

6.	Farris JS. Phylogenetic Analysis Under Dollo’s Law. Syst Zool. 1977 Mar;26(1):77. doi:10.2307/2412867

7.	Eddy SR. Accelerated Profile HMM Searches. Pearson WR, editor. PLoS Comput Biol. 2011 Oct;7(10):e1002195. doi:10.1371/journal.pcbi.1002195

8.	Jones P, Binns D, Chang HY, Fraser M, Li W, McAnulla C, et al. InterProScan 5: genome-scale protein function classification. Bioinformatics. 2014 Jan;30(9):1236–40. doi:10.1093/bioinformatics/btu031

9.	Seemann T. Prokka: rapid prokaryotic genome annotation. Bioinformatics. 2014 Mar;30(14):2068–9. doi:10.1093/bioinformatics/btu153

10.	Cantalapiedra CP, Hernández-Plaza A, Letunic I, Bork P, Huerta-Cepas J. eggNOG-mapper v2: Functional Annotation, Orthology Assignments, and Domain Prediction at the Metagenomic Scale. Tamura K, editor. Mol Biol Evol. 2021 Oct;38(12):5825–9. doi:10.1093/molbev/msab293

11.	Lin Z, Akin H, Rao R, Hie B, Zhu Z, Lu W, et al. Evolutionary-scale prediction of atomic-level protein structure with a language model. Science. 2023 Mar;379(6637):1123–30. doi:10.1126/science.ade2574

12.	van Kempen M, Kim SS, Tumescheit C, Mirdita M, Lee J, Gilchrist CLM, et al. Fast and accurate protein structure search with Foldseek. Nat Biotechnol. 2024;42(2):243–6. doi:10.1038/s41587-023-01773-0

13.	Mirdita M, Schütze K, Moriwaki Y, Heo L, Ovchinnikov S, Steinegger M. ColabFold: making protein folding accessible to all. Nat Methods. 2022 May;19(6):679–82. doi:10.1038/s41592-022-01488-1

14.	Jumper J, Evans R, Pritzel A, Green T, Figurnov M, Ronneberger O, et al. Highly accurate protein structure prediction with AlphaFold. Nature. 2021 Jul;596(7873):583–9. doi:10.1038/s41586-021-03819-2

15.	Diener C, Gibbons SM, Resendis-Antonio O. MICOM: Metagenome-Scale Modeling To Infer Metabolic Interactions in the Gut Microbiota. mSystems. 2020 Jan 21;5(1):e00606-19. doi:10.1128/mSystems.00606-19 PubMed PMID: 31964767; PubMed Central PMCID: PMC6977071.

16.	Rancurel C, Legrand L, Danchin EGJ. Alienness: Rapid Detection of Candidate Horizontal Gene Transfers across the Tree of Life. Genes. 2017 Sep 29;8(10):248. doi:10.3390/genes8100248 PubMed PMID: 28961181; PubMed Central PMCID: PMC5664098.

17.	Buchfink B, Reuter K, Drost HG. Sensitive protein alignments at tree-of-life scale using DIAMOND. Nat Methods. 2021 Apr;18(4):366–8. doi:10.1038/s41592-021-01101-x

18.	Capella-Gutiérrez S, Silla-Martínez JM, Gabaldón T. trimAl: a tool for automated alignment trimming in large-scale phylogenetic analyses. Bioinformatics. 2009 Jun;25(15):1972–3. doi:10.1093/bioinformatics/btp348

19.	Kalyaanamoorthy S, Minh BQ, Wong TKF, von Haeseler A, Jermiin LS. ModelFinder: fast model selection for accurate phylogenetic estimates. Nat Methods. 2017 May;14(6):587–9. doi:10.1038/nmeth.4285

20.	Lovley DR. Electrically conductive pili: Biological function and potential applications in electronics. Curr Opin Electrochem. 2017 Aug 1;4(1):190–8. doi:10.1016/j.coelec.2017.08.015

21.	Walker DJF, Adhikari RY, Holmes DE, Ward JE, Woodard TL, Nevin KP, et al. Electrically conductive pili from pilin genes of phylogenetically diverse microorganisms. ISME J. 2018;12(1):48–58. doi:10.1038/ismej.2017.141

22.	Bray MS, Wu J, Padilla CC, Stewart FJ, Fowle DA, Henny C, et al. Phylogenetic and structural diversity of aromatically dense pili from environmental metagenomes. Environ Microbiol Rep. 2020;12(1):49–57. doi:10.1111/1758-2229.12809

23.	Walker DJF, Nevin KP, Holmes DE, Rotaru AE, Ward JE, Woodard TL, et al. *Syntrophus* conductive pili demonstrate that common hydrogen-donating syntrophs can have a direct electron transfer option. ISME J. 2020;14(3):837–846. doi:10.1038/s41396-019-0575-9

24.	Chan SHJ, Simons MN, Maranas CD. SteadyCom: Predicting microbial abundances while ensuring community stability. Price ND, editor. PLoS Comput Biol. 2017 May;13(5):e1005539. doi:10.1371/journal.pcbi.1005539

25.	de Bok FAM, Stams AJM, Dijkema C, Boone DR. Pathway of Propionate Oxidation by a Syntrophic Culture of *Smithella* *propionica* and Methanospirillum hungatei. Appl Environ Microbiol. 2001 Apr;67(4):1800–4. doi:10.1128/aem.67.4.1800-1804.2001

26.	Dolfing J. Syntrophic Propionate Oxidation via Butyrate: a Novel Window of Opportunity under Methanogenic Conditions. Appl Environ Microbiol. 2013 Jul;79(14):4515–6. doi:10.1128/AEM.00111-13

27.	Thauer RK, Jungermann K, Decker K. Energy conservation in chemotrophic anaerobic bacteria. Bacteriol Rev. 1977 Mar;41(1):100–80. doi:10.1128/br.41.1.100-180.1977

28. Paradis E, Schliep K. ape 5.0: an environment for modern phylogenetics and evolutionary analyses in R. Bioinformatics. 2019;35(3):526–8. doi:10.1093/bioinformatics/bty633

29. Revell LJ. phytools: an R package for phylogenetic comparative biology (and other things). Methods Ecol Evol. 2012;3(2):217–23. doi:10.1111/j.2041-210X.2011.00169.x
